# Multidimensional Machine Learning for Assessing Parameters Associated With COVID-19 in Vietnam: Validation Study

**DOI:** 10.2196/42895

**Published:** 2023-02-16

**Authors:** Trong Tue Nguyen, Cam Tu Ho, Huong Thi Thu Bui, Lam Khanh Ho, Van Thanh Ta

**Affiliations:** 1 Medical Laboratory Department Hanoi Medical University Hanoi Vietnam; 2 Clinical Laboratory Department Hanoi Medical University Hospital Hanoi Vietnam; 3 Center of Gene and Protein Research Hanoi Medical University Hanoi Vietnam; 4 Institute of Virology TUM School of Medicine Technical University of Munich Munich Germany; 5 Biochemistry Department Thai Nguyen University of Medicine and Pharmacy Thai Nguyen Vietnam; 6 Faculty of Information Technology Hung Yen University of Technology and Education Hung Yen Vietnam

**Keywords:** COVID-19, multidimensional analysis, hierarchical cluster analysis, regression analysis, mild, moderate, severe, age, scoring index of chest x-ray, percentage and quantity of neutrophils, albumin, C-reactive protein, ratio of lymphocytes

## Abstract

**Background:**

Machine learning (ML) is a type of artificial intelligence strategy. Its algorithms are used on big data sets to see patterns, learn from their results, and perform tasks autonomously without being instructed on how to address problems. New diseases like COVID-19 provide important data for ML. Therefore, all relevant parameters should be explicitly quantified and modeled.

**Objective:**

The purpose of this study was to determine (1) the overall preclinical characteristics, (2) the cumulative cutoff values and risk ratios (RRs), and (3) the factors associated with COVID-19 severity in unidimensional and multidimensional analyses involving 2173 SARS-CoV-2 patients.

**Methods:**

The study population consisted of 2173 patients (1587 mild status [mild group] and asymptomatic patients, 377 moderate status patients [moderate group], and 209 severe status patients [severe group]). The status of the patients was recorded from September 2021 to March 2022. Two correlation tests, relative risk, and RR were used to eliminate unbalanced parameters and select the most remarkable parameters. The independent methods of hierarchical cluster analysis and k-means were used to classify parameters according to their *r* values. Finally, network analysis provided a 3-dimensional view of the results.

**Results:**

COVID-19 severity was significantly correlated with age (mild-moderate group: RR 4.19, 95% CI 3.58-4.95; *P*<.001), scoring index of chest x-ray (mild-moderate group: RR 3.29, 95% CI 2.76-3.92; *P*<.001; moderate-severe group: RR 3.03, 95% CI 2.4023-3.8314; *P*<.001), percentage of neutrophils (mild-moderate group: RR 3.18, 95% CI 2.73-3.70; *P*<.001; moderate-severe group: RR 3.32, 95% CI 2.6480-4.1529; *P*<.001), quantity of neutrophils (moderate-severe group: RR 3.15, 95% CI 2.6153-3.8025; *P*<.001), albumin (moderate-severe group: RR 0.46, 95% CI 0.3650-0.5752; *P*<.001), C-reactive protein (mild-moderate group: RR 3.4, 95% CI 2.91-3.97; *P*<.001), and ratio of lymphocytes (moderate-severe group: RR 0.34, 95% CI 0.2743-0.4210; *P*<.001). Significant inversion of correlations among the severity groups is important. Alanine transaminase and leucocytes showed a significant negative correlation (*r*=−1; *P*<.001) in the mild group and a significant positive correlation in the moderate group (*r*=1; *P*<.001). Transferrin and anion Cl showed a significant positive correlation (*r*=1; *P*<.001) in the mild group and a significant negative correlation in the moderate group (*r*=−0.59; *P*<.001). The clustering and network analysis showed that in the mild-moderate group, the closest neighbors of COVID-19 severity were ferritin and age. C-reactive protein, scoring index of chest x-ray, albumin, and lactate dehydrogenase were the next closest neighbors of these 3 factors. In the moderate-severe group, the closest neighbors of COVID-19 severity were ferritin, fibrinogen, albumin, quantity of lymphocytes, scoring index of chest x-ray, white blood cell count, lactate dehydrogenase, and quantity of neutrophils.

**Conclusions:**

This multidimensional study in Vietnam showed possible correlations between several elements and COVID-19 severity to provide clinical reference markers for surveillance and diagnostic management.

## Introduction

The COVID-19 pandemic has become one of the most severe health crises in human history, spreading rapidly across the globe since January 2020. The outbreak has been reported in over 200 countries globally. Vietnam had the first 16 typical cases confirmed positive up to February 28, 2020. After thoroughly applying medical prevention and active control strategies, Vietnam took control of the COVID-19 outbreak according to a recent World Health Organization assessment [1]. Vietnam has been reported as an influential country in preventing and controlling the COVID-19 outbreak [2]. We performed a multidimensional study of 2173 hospitalized COVID-19 patients from September 2021 to March 2022 at the Hospital of Hanoi Medical University in Vietnam. All of these patients had a SARS-CoV-2 polymerase chain reaction cycle threshold (Ct) of <30 cycles. We obtained all medical records at the first diagnosis of COVID-19, and all data in digital medical records were collected.

Machine learning (ML) provides methods, techniques, and tools to help solve diagnostic and prognostic problems in various medical domains. In recent years, ML algorithms have been more widely and increasingly applied in biomedical fields, for example, in autism spectrum disorder or asthma research, or in several steps of hospital management, such as the emergency department [3-5]. It has been used to analyze the importance of clinical parameters and their combinations for prognosis (eg, prediction of disease progression, extraction of medical knowledge for outcome research, therapy planning and support, and overall patient management). ML is also essential for data analysis, such as detecting regularities in the data by appropriately dealing with imperfect data, interpreting continuous data used in the intensive care unit, and intelligent alarm management, resulting in effective and efficient monitoring. Medical diagnostic reasoning is a critical application area of intelligent systems [6,7]. ML techniques are based on algorithms, which are sets of mathematical procedures that describe the relationships between variables. The purpose of this study was to determine (1) the overall preclinical characteristics, (2) the cumulative cutoff values and risk ratios (RRs), and (3) the factors associated with COVID-19 severity in unidimensional and multidimensional analyses involving 2173 SARS-CoV-2 patients.

## Methods

### Ethics Approval

This study is part of the project “Building a Database of Genomic Variants of SARS-CoV-2 in Vietnam” (study code: DTDLCN.102/21, MOST VIETNAM), which was recognized and approved by the Institutional Review Board for Ethics in Biomedical Research of Hanoi Medical University (code: IRB-VN01001/IRB00003121/FWA00004148, number 546/GCN-HDDDNCYSH-DHYHN). All participants’ rights were protected, and informed consent was obtained from the participants.

### Study Population

Following the guidance for the clinical management of COVID-19 from the World Health Organization (November 23, 2021) and the latest advice on the diagnosis and treatment of COVID-19 from the Ministry of Health (article number 250/QD-BYT) [8], an asymptomatic group was added to the classification of COVID-19 disease severity, in addition to the 4 groups of mild, moderate, severe, and critical. In our study, there were 1098 mild patients, 386 moderate patients, and 203 severe patients.

The mild group included COVID-19 patients who had nonspecific clinical symptoms, such as fever, dry cough, sore throat, nasal congestion, fatigue, headache, muscle pain, loss of taste, loss of smell, diarrhea, etc. Moreover, these patients had a breathing rate of <20 breaths/minute and SpO_2_ of >96% when breathing air. Furthermore, these patients were awake and could perform activities on their own. Chest radiography indicated standard findings or minimal damage.

Inclusion in the moderate group was determined on assessing the overall condition. This group included patients with nonspecific clinical symptoms such as mild severity. Regarding respiration, patients showed signs of pneumonia, shortness of breath, rapid breathing (20-25 breaths/minute), lung crackles, and SpO_2_ of 94%-96% when breathing room air, with no signs of severe respiratory failure. There may be difficulty breathing on exertion (walking indoors and climbing stairs). Moreover, patients may have a fast or slow pulse, dry skin, tachycardia, normal blood pressure, and alert consciousness. In addition, chest radiography and chest computed tomography detected lesions (<50%), and ultrasound showed B waves. Arterial blood gas analysis indicated PaO_2_/FiO_2_ >300.

The severe group included patients with respiratory signs of pneumonia accompanied by any of the following: respiratory rate >25 breaths/minute, severe shortness of breath, contraction of accessory respiratory muscles, and SpO_2_ of <94% when breathing room air. Regarding circulation, patients may have a fast or slow heart rate, and normal or increased blood pressure. Neurologically, patients may be restless or lethargic and tired. Chest radiography and chest computed tomography detected lesions (>50%), and ultrasound showed many B waves. Arterial blood gas analysis indicated PaO_2_/FiO_2_ of about 200-300.

### ML: Multidimensional and Stratified Analysis

**Figure 1:**
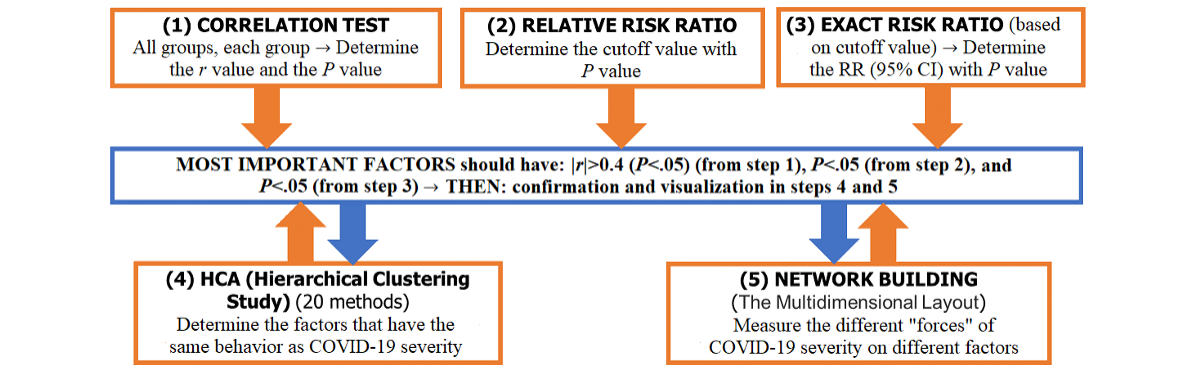
Multidimensional machine learning approach. RR: risk ratio.

The data collection, storage, and analysis were conducted in R 4.1.0 (R Project for Statistical Computing). Our data set for multidimensional analysis included medical test parameters for 2713 COVID-19 patients with 3 types of COVID-19 status (mild, moderate, and severe). This was considered as multivariate statistics, which is a subdivision of statistics encompassing the simultaneous observation and analysis of more than one outcome variable. Multivariate statistics concerns understanding the different aims and backgrounds of each of the different forms of multivariate analysis and how they relate to each other. For our multidimensional analysis, we used several algorithms, including regularized general linear model regression, hierarchical cluster analysis (HCA), and artificial network contribution [9]. The multidimensional analysis provided the index of dimension objects organized in meaningful hierarchies (the ordered series of related dimensions). It allowed us to observe data from various viewpoints and enabled us to spot trends or exceptions in the data (Figure 1).

The general linear model is a model involving a single normal distribution, where we are interested in statements about the mean of that distribution. The resulting test is known as the 1-sample *t* test. The independent sample *t* test by 1-way ANOVA, Pearson chi-square test, and Fisher exact test were used to clarify the differences (Figure 1).

The correlation *r* measures the strength of the linear relationship between 2 quantitative variables, and the value of *r* is between −1 and 1. An *r* value >0 indicates a positive association, and a value <0 indicates a negative association. Values of *r* near 0 indicate a fragile linear relationship. The strength of the linear affinity increases as *r* moves away from 0 toward −1 or 1. The extreme *r* values of −1 and 1 occur only in the case of a perfect linear relationship [10].

In our meta-analysis, the cutoff values, RRs, odds ratios, and 95% CIs were calculated by the regression analysis, and the significant factors were selected for HCA, k-means, and network studies in R 4.1.0 environment, which were performed based on the correlation test results. HCA is a concept of cluster analysis that builds a hierarchy of clusters called a dendrogram. In the HCA process, the first step is to calculate the distance between each observation, and the second step is to estimate the cluster’s distance to the remaining statements. If the shortest distance is between 2 variables, a second cluster is defined; otherwise, the observation is added as a new level to the set. The process repeats until all statements belong to a single set. The linkage mode of “distance” to a cluster can be determined as follows: complete mode (distance to the furthest member of the cluster), single mode (spread to the closest member of the cluster), average mode (average length to all members of the group), ward (minimizes the total within-cluster variable), mcquitty (the distance between clusters is the weighted mean of the between-cluster dissimilates), or centroid (space between the centroids of each cluster). In our study, we used the mode of complete linkage to calculate the Euclidean distance and classify the cluster to build the dendrogram. Complete and average distances produce more balanced trees, which are the most common and helpful for identifying outliers. The vertical lines in a dendrogram indicate the distance between nodes and their associated cluster. In the dendrogram, each leaf corresponds to 1 object. As we move up the tree, objects similar to each other combine into branches fused at a higher height. We verified how well the cluster tree was generated by computing the correlation between the cophenetic distances and the initial distance data generated. If the clustering is valid, the objects’ connections in the cluster tree should strongly correlate with their lengths in the original distance matrix. The closer the correlation coefficient is to 1, the more accurately the clustering reflects the data in reality.

The results of HCA will provide the first view of the arrangement of our data. It is important to choose the number of clusters to keep following k-means by identifying a cut point that creates a reasonable number of sets with a substantial number of observations per cluster. The values of the indexes can be independently used to determine the number of clusters existing in a data set. However, only the following 3 indices have been used: silhouette, gap, and within-cluster sum of square. The k-means method calculates the values of several clustering indexes. Thus, we combined 2 methods to obtain a better number of clusters. K-means is a reallocation method. The first step is to select as many points as the number of clusters to create initial centers. Then, each observation is associated with the nearest center to create temporary clusters. The next step is to look for the gravity centers of each temporary cluster, and these become the new cluster centers. Finally, each observation is reallocated to the cluster that has the closest center. This procedure is iterated until convergence. K-means computation was optimized following 20 indexes. These results were used to conduct the principal component analysis (PCA) cluster plot, in which we visualized the best cluster number.

Network analysis is a visualization approach that can represent various types of data. Several models can be used to build the map of nodes with their force of influence on each other. One of them is the layout called multidimensional scaling (MDS). MDS is a means of visualizing the level of similarity of individual cases of a data set.

MDS is used to decode the pairwise distances among a set of *n* objects or individuals into a layout of *n* points mapped to a conceptual Cartesian distance. MDS refers to a set of corresponding ordination techniques used in information visualization to display the information in a distance matrix. It is a structure of nonlinear dimensionality reduction, which is given in a distance matrix with the distances between each pair of objects in a set and a chosen *N* number of dimensions. An MDS algorithm sets each object in an N-dimensional area to preserve the between-object distances as much as possible. With N=1, 2, and 3, the resulting points can be visualized on a scatter plot [11].

The MDS layout provides vision of the COVID-19 outcome influence on other factors and how the link was disturbed by other neighbors. Based on the *r* values, the data to be analyzed is a collection of *M* objects (in our study, it will be the number of preclinical factors in each imputation) on which a distance function defines the entries of the dissimilarity matrix. The distance function from the multidimensional layout is as follows [11]:



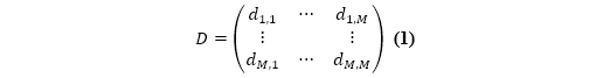



where *d_i,j_* is the distance between the *i*th and *j*th objects.

## Results

### Overview of the Cohort

A total of 2173 COVID-19 patients were included in our study. Of these 2173 patients, 1587 had mild symptoms or no symptoms, 377 had moderate symptoms, and 209 had severe symptoms. Considering all collected information, we found significant differences in several factors between the severity groups. All patient information regarding medical history and cause of hospitalization was collected and analyzed. Medical history was recorded and classified according to the International Classification of Diseases 10th Revision (ICD-10) list of the Ministry of Health of Vietnam. The ANOVA test showed a statistically significant difference between the distribution of medical history and the severity of COVID-19. No correlation was found between COVID-19 severity and patient medical history (Multimedia Appendix 1).

The classification of COVID-19 patients was based on the guidelines of the Ministry of Health, in which the SpO_2_ index was used as the main criterion. A sudden decrease in SpO_2_ is believed to be the hallmark of a worsening patient. The parameters of COVID-19 severity were provided by our colleagues (ie, doctors at the Field Hospital-Hanoi Medical University Hospital) for a suitable treatment regimen in each patient. A total of 66 subclinical indicators were separated for better analysis. There was a significant difference in age between the mild group and the moderate and severe groups (*P*<.001). Similar results were obtained for aspartate transaminase (AST), alanine transaminase (ALT), hemoglobin, mean corpuscular hemoglobin, quantity of monocytes, and COVID-19 vaccine boosters. When we compared the groups (mild vs moderate group, moderate vs severe group, and mild vs severe group), there were differences in several factors, including urea, pH, scoring index of chest x-ray, fibrinogen, percentage and quantity of monocytes, number of lymphocytes, percentage of basophils, red blood cell count, white blood cell (WBC) count, ratio of lymphocytes, quantity and percentage of neutrophils, anion Cl, pCO_2_, SpO_2_, D-dimer, glucose, albumin, transferrin, ferritin, C-reactive protein (CRP), pro–B-type natriuretic peptide (pro-BNP), troponin T, creatinine, and lactate dehydrogenase (LDH). There were significant differences in some factors (eg, number of basophils, beta-adrenergic blockers, ratio of prothrombin, time and ratio of activated partial thromboplastin time, leucocytes, and specific gravity) between the mild/moderate group and severe group. Total bilirubin showed a significant difference between the mild and moderate groups (Multimedia Appendix 1).

### Correlation Between COVID-19 Severity and 67 Preclinical Factors

**Figure 2:**
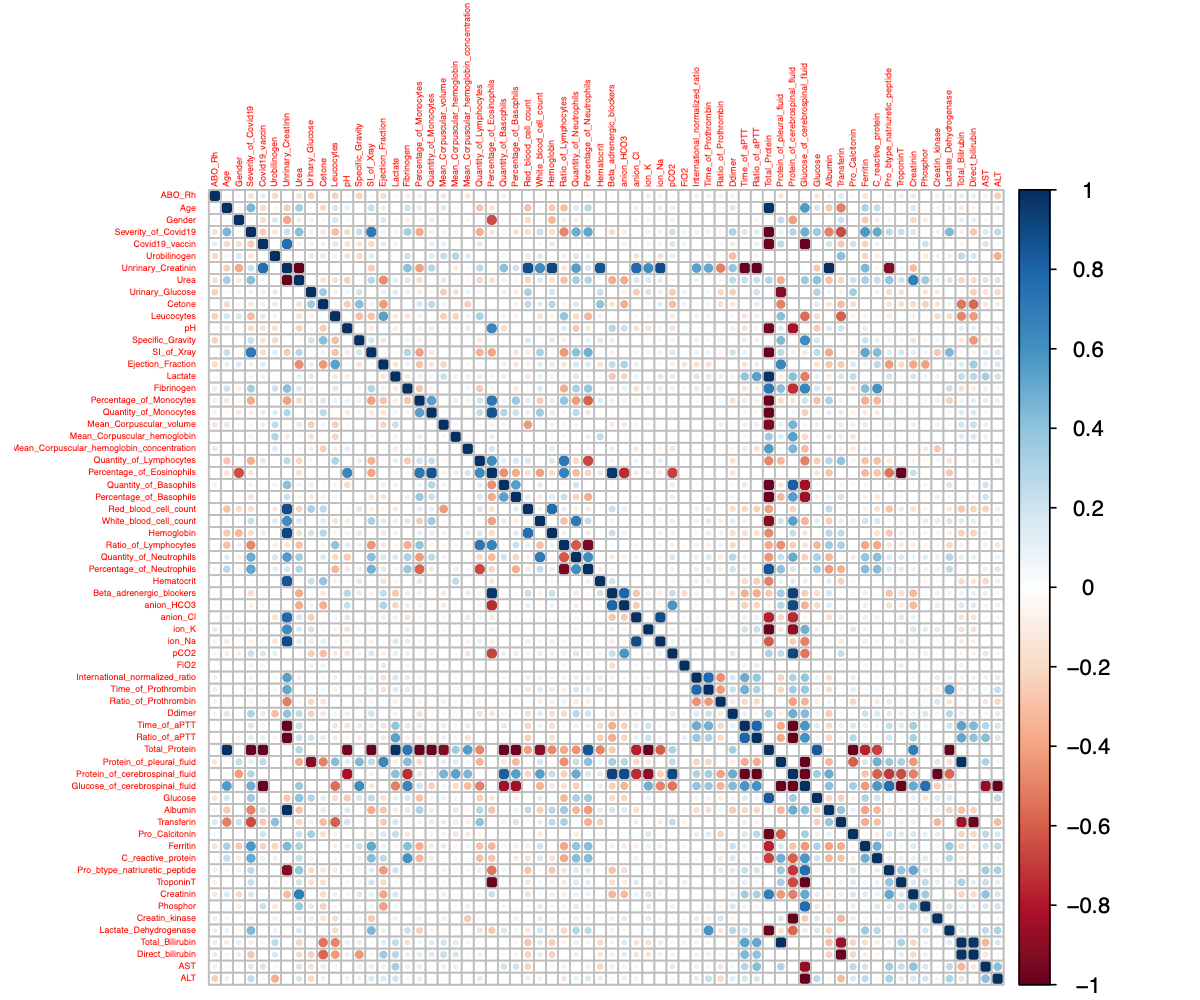
Correlation matrix between biomarkers depicted as a heat map. The heat map presents the color-coded correlations among 67 preclinical factors. The color intensity of the cells is proportional to the strength of the correlation, ranging from red (negative correlation) to blue (positive correlation). The strength of the correlation is indicated in the color scale (right of the panel). Pairwise Pearson correlation coefficients are shown in Multimedia Appendices 2 and 3.

Using the electronic medical records of 2173 COVID-19 patients, we calculated the *r* values, which showed the direction of correlation between the assessed factors (Figure 2; Multimedia Appendix 2 and Multimedia Appendix 3). Figure 2 shows the general view of some strong associations in both directions (negative and positive). We focused on the association of COVID-19 severity with all factors. Severity was strongly positively correlated with age (*r*=0.45), urea (*r*=0.43), scoring index of chest x-ray (*r*=0.71), quantity of neutrophils (*r*=0.50), ratio of neutrophils (*r*=0.52), quantity of protein (*r*=0.29) and glucose (*r*=0.49) in the cerebrospinal fluid, quantity of glucose in the blood (*r*=0.37), ferritin (*r*=0.58), CRP (*r*=0.50), and LDH (*r*=0.44) (all *P*<.001). On the other hand, severity was strongly negatively correlated with albumin (*r*=−0.50), transferrin (*r*=−0.65), and SpO_2_ (*r*=−0.39) (all *P*<.001) (Figure 2; Multimedia Appendix 2 and Multimedia Appendix 3).

**Figure 3:**
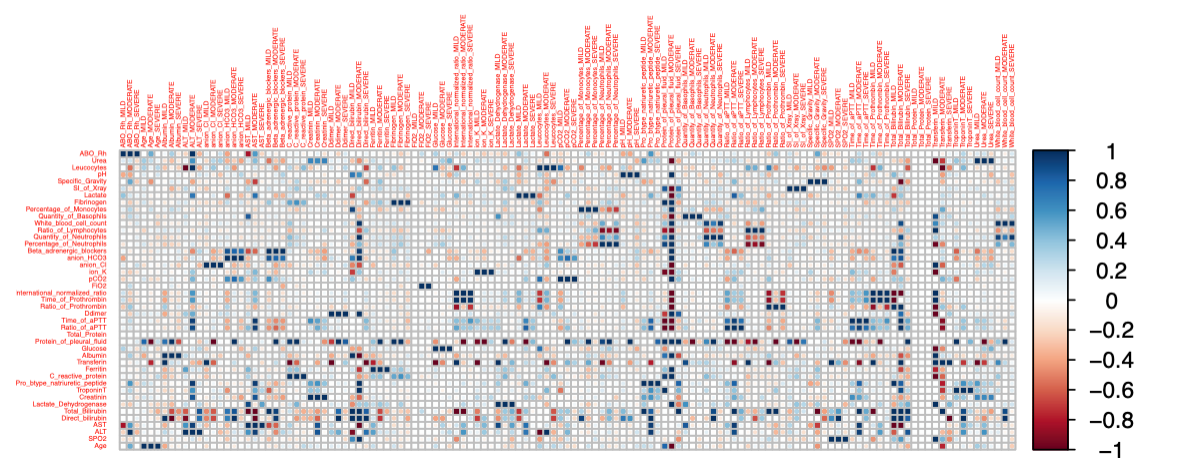
Correlation matrix between biomarkers depicted as a heat map. The heat map presents the color-coded correlations among 43 preclinical factors in the 3 disease severity groups. The color intensity of the cells is proportional to the strength of the correlation, ranging from red (negative correlation) to blue (positive correlation). The strength of the correlation is indicated in the color scale (right of the panel). Pairwise Pearson correlation coefficients are shown in Multimedia Appendices 4 and 5.

We divided the 2173 patients into the following groups: mild and no symptom group (1093 and 494 patients, respectively), moderate group (377 patients), and severe group (209 patients). Considering all collected information, we found significant differences in several factors and a critical shift in the association direction among the severity groups (Figure 3; Multimedia Appendices 4 and 5).

We selected 43 common factors in the 3 groups to run the second correlation test, and we found different correlative indications. Multimedia Appendix 5 shows all the pairs that had not only a significant association but also an inverse correlation according to COVID-19 severity. Their absolute *r* values ranged from 0.20 to 1 (Figure 3; Multimedia Appendix 5-Multimedia Appendix 11).

We included all the *r* values from the 3 groups in a pairwise *t* test. We again found significant differences (*P*<.05) in several factors, which confirmed an important shift in the correlation among the groups of COVID-19 severity (Multimedia Appendix 12).

### Cutoff Values and Exact RRs Showing the Effect of the Predicted Values on COVID-19 Severity

We used a logistic regression model to predict subclinical values that are associated with a 50:50 probability that the status of a mild/moderate disease patient could be severe (Multimedia Appendix 13). Several factors had a significant cutoff_50_ (*P*<.05), which is the boundary between mild/moderate disease and severe disease. Considering these values in the meta-RR analysis, using several meta-analytical methods, we found an RR value of 1.493 (95% CI 1.42-1.57; z=16.12; *P*<.001) (Table 1). We noted very high RR values ranging from 2.47 to 6.18 for the following factors: pro-BNP, number of basophils, troponin T, FiO_2_, ferritin, glucose, urea, D-dimer, CRP, scoring index of chest x-ray, WBC count, anion Cl, ion K, ratio and quantity of neutrophils, and LDH. Other factors, such as ratio of lymphocytes, direct and total bilirubin, albumin, transferrin, pH, protein in the pleural fluid, and SpO_2_, showed very low RR values (0.17 to 0.48), which fits with their inverse correlation with COVID-19 severity.

More details were obtained when taking 2 groups together for the relative RR imputation and the exact RR. The meta-RR analysis showed that the pooled RR in the mild-moderate group was 2.78 (95% CI 2.66-2.90; z=45.29; *P*<.001) and the pooled RR in the moderate-severe group was 1.28 (95% CI 1.22-1.33; z=11.23; *P*<.001) (Multimedia Appendix 13). We selected the *r* value (|*r*|>0.3) according to COVID-19 severity to calculate the RR. The percentage of neutrophils, CRP, ferritin, age, and scoring index of chest x-ray showed strong and significant correlations with COVID-19 severity (mild and moderate; *r* values of 0.33, 0.40, 0.43, 0.44, and 0.52, respectively; all *P*<.001), and their RR values for the cutoff_50_ values were significantly higher than the pooled RR (RR values of 3.18, 3.4, 3.62, 4.19, and 3.29, respectively; all *P*<.001).

In the moderate-severe group, percentage and number of neutrophils, and scoring index of chest x-ray showed significant positive correlations with COVID-19 severity (RR values of 3.32, 3.15, and 3.03, respectively; all *P*<.001), and the RR values were higher than the pooled RR. Albumin and ratio of lymphocytes had powerful negative correlations with COVID-19 severity (RR values of 0.46 and 0.34, respectively; all *P*<.001), and the RR values were lower than the pooled RR (Multimedia Appendix 14 and Multimedia Appendix 15).

**Table 1:** Risk ratio data of 41 preclinical factors.

Preclinical factor^a^	Risk ratio (95% CI)	%W (common)
Ratio of lymphocytes	0.1700 (0.1355-0.2132)	12.9
SpO_2_	0.1956 (0.1565-0.2444)	8.0
Direct bilirubin	0.3191 (0.0264-3.8550)	0.3
Total bilirubin	0.3767 (0.0310-4.5715)	0.3
Albumin	0.3937 (0.3107-0.4988)	10.4
Transferrin	0.4375 (0.1594-1.2006)	1.0
pH	0.4725 (0.3692-0.6047)	6.7
Protein in the pleural fluid	0.4839 (0.2894-0.8091)	0.2
Beta-adrenergic blockers	0.5120 (0.4114-0.6373)	9.0
Anion HCO_3_	0.5416 (0.4122-0.7116)	5.0
Total protein	0.5556 (0.0466-6.6286)	0.1
Leucocytes	0.5814 (0.2734-1.2366)	1.2
Percentage of monocytes	0.6117 (0.3254-1.1499)	1.4
International normalized ratio	0.9464 (0.4871-1.8387)	1.1
Time of prothrombin	0.9994 (0.5435-1.8379)	1.2
Ratio of prothrombin	1.1051 (0.5925-2.0612)	1.2
Specific gravity	1.3002 (0.9030-1.8720)	2.8
Time of aPTT^b^	1.3843 (1.0916-1.7555)	4.9
Ratio of aPTT	1.4076 (1.1098-1.7853)	4.9
Aspartate transaminase	1.5357 (0.6769-3.4840)	0.3
Creatinine	1.6515 (0.5951-4.5832)	0.3
Alanine transaminase	1.9385 (1.0739-3.4990)	0.6
Fibrinogen	1.9451 (0.9649-3.9211)	0.5
Lactate	2.0724 (1.2316-3.4874)	0.5
pCO_2_	2.3810 (1.9092-2.9693)	3.0
Pro–B-type natriuretic peptide	2.4669 (1.8245-3.3354)	1.6
Quantity of basophils	2.6135 (0.5248-13.0144)	0.1
Troponin T	2.6841 (2.0363-3.5380)	1.6
FiO_2_	3.2801 (2.9251-3.6782)	0.2
Ferritin	3.3031 (2.4481-4.4567)	1.9
Glucose	3.4376 (2.2348-5.2877)	0.7
Urea	3.7561 (2.8322-4.9813)	1.6
D-dimer	4.4144 (3.2591-5.9792)	1.0
C-reactive protein	4.4427 (3.1382-6.2894)	0.9
Scoring index of chest x-ray	4.8518 (3.7883-6.2138)	2.9
White blood cell count	5.1577 (3.9121-6.8000)	0.9
Anion Cl	5.2427 (2.3317-11.7882)	0.1
Ion K	5.4388 (3.0428-9.7216)	0.1
Percentage of neutrophils	5.5665 (4.4259-7.0012)	2.4
Lactate dehydrogenase	5.8781 (4.5904-7.5270)	1.3
Quantity of neutrophils	6.1821 (4.9578-7.7087)	1.7

^a^In meta-analytical methods, such as the Mantel-Haenszel method, restricted maximum likelihood estimator for *τ*^2^, Q-profile method for the CI of *τ*^2^ and *τ*, and continuity correction of 0.5 in studies with zero cell frequencies, we found: risk ratio=1.39 (95% CI 1.33-1.46; z=13.73; *P*<.001), *τ*^2^=0.9682 (95% CI 0.6084-1.5641), *τ*=0.9840 (95% CI 0.7800-1.2506), *I*^2^=98.0% (95% CI 97.7%-98.3%), and H=7.05 (95% CI 6.58-7.56). For test of heterogeneity, the findings were as follows: Q=2040.66; *df*=41; *P*<.001.

^b^aPTT: activated partial thromboplastin time.

### HCA and K-means Clustering

**Figure 4:**
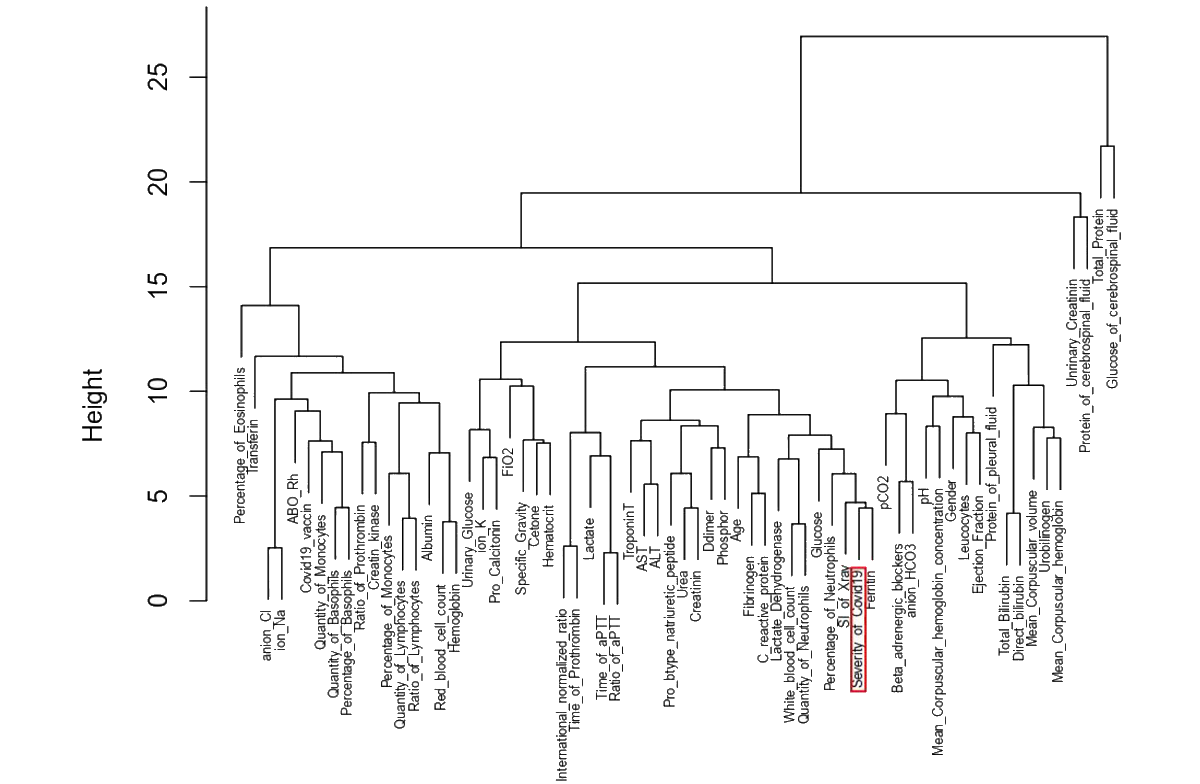
Cluster dendrogram of all 3 patient groups (66 parameters from the correlation test; details are in Figure 2). The linkage model is “complete.” The height axis displays the Euclidean distance between observations or clusters. The horizontal bars indicate when 2 clusters/observations merge.

**Figure 5:**
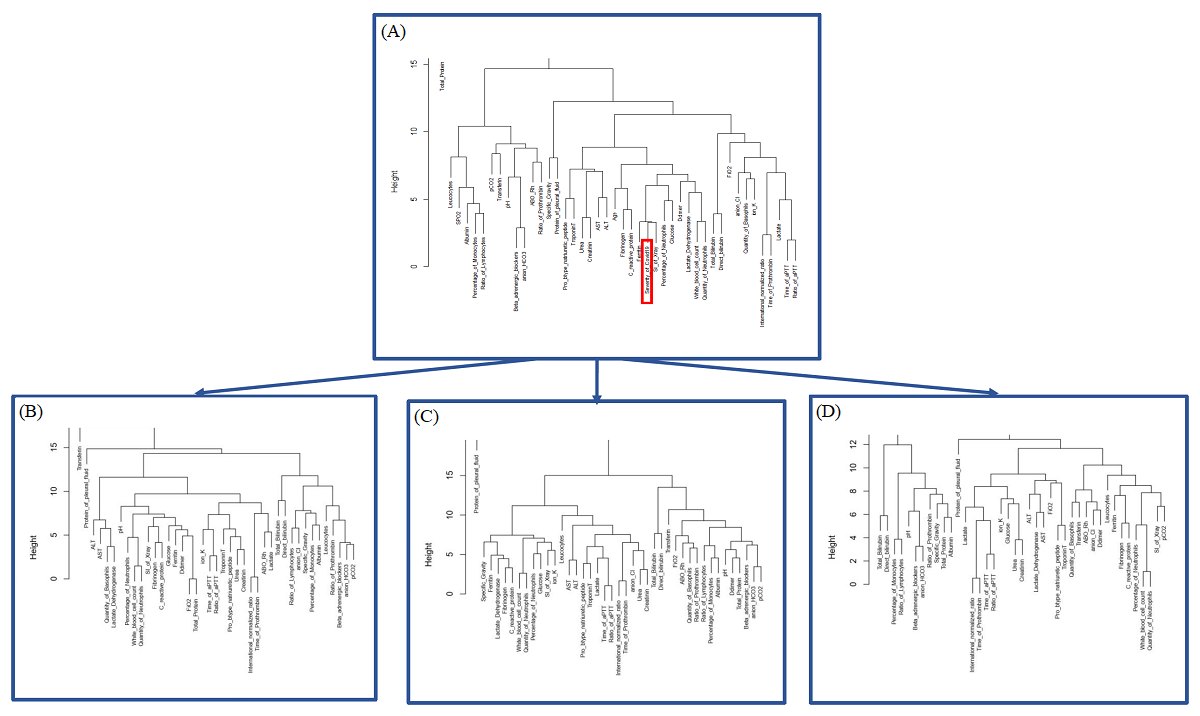
Cluster dendrogram after the second correlation test and the relative risk and risk ratio imputation (details are in Figure 3, Multimedia Appendices 13, and 14). The linkage model is “complete.” (A) All 3 patient groups (42+1 parameters from the second correlation test; details are in Figure 3). (B) Mild group (41 parameters selected from the relative risk and risk ratio *P* value; details are in Multimedia Appendices 13 and 14). (C) Moderate group (41 parameters selected from the relative risk and risk ratio *P* value; details are in Multimedia Appendices 13 and 14). (D) Severe group (41 parameters selected from the relative risk and risk ratio *P* value; details are in Multimedia Appendices 13 and 14). The height axis displays the Euclidean distance between observations or clusters. The horizontal bars indicate when 2 clusters/observations merge.

Based on the correlation (r) identified, clustering imputation showed the difference in branching between factors. Consequently, the resulting clustering scheme required some evaluation regarding its validity [12]. The dendograms were built based on the clustering metric “Euclidean” and the method “complete,” in which we found the following 9 neighboring factors around COVID-19 severity: D-dimer, fibrinogen, CRP, LDH, WBC count, number of neutrophils, glucose, scoring index of chest x-ray, and ferritin (Figures 4 and 5). The correlation between cophenetic distances and the original distance data generated showed that the most appropriate linkage model was “average,” and the “complete” method showed medium correlation (Table 2).

The neighbors of these 9 factors changed in the 3 disease groups (mild, moderate, and severe). In the severe group, glucose and LDH split into other groups. Their behaviors with regard to severity changed. However, HCA failed to define the number of clusters in our data set for the following 3 proposed methods: gap statistic, elbow, and silhouette. We need to consider other methods to observe more classification possibilities.

**Figure 6:**
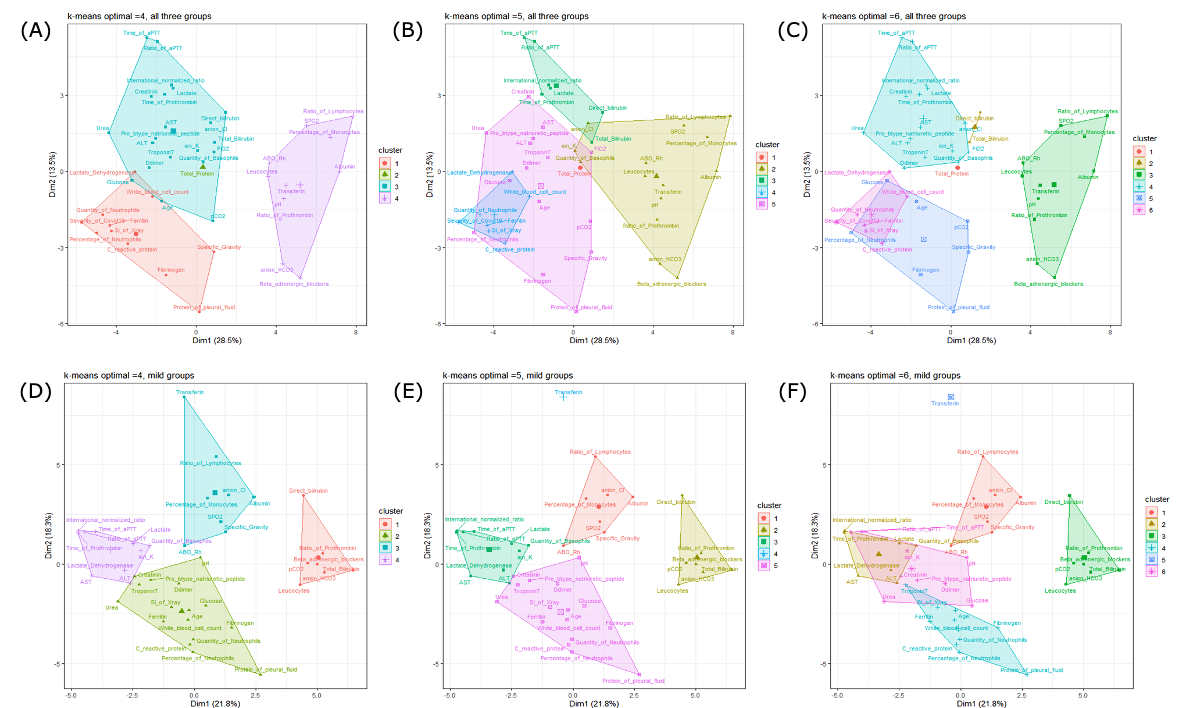
Principal component analysis cluster plot of all 3 groups (A-C) and the mild group (D-F) in 2-dimensional space (dim1 and dim2). These dimensions can be interpreted as principal components (PCs). In all 3 groups, the PCs accounted for 42.0% (13.5% [dim1] + 28.5% [dim2]) of the total variation. In the mild group, the PCs accounted for 40.1% (21.8% [dim1] + 18.3% [dim2]) of the variation in the data set.

**Figure 7:**
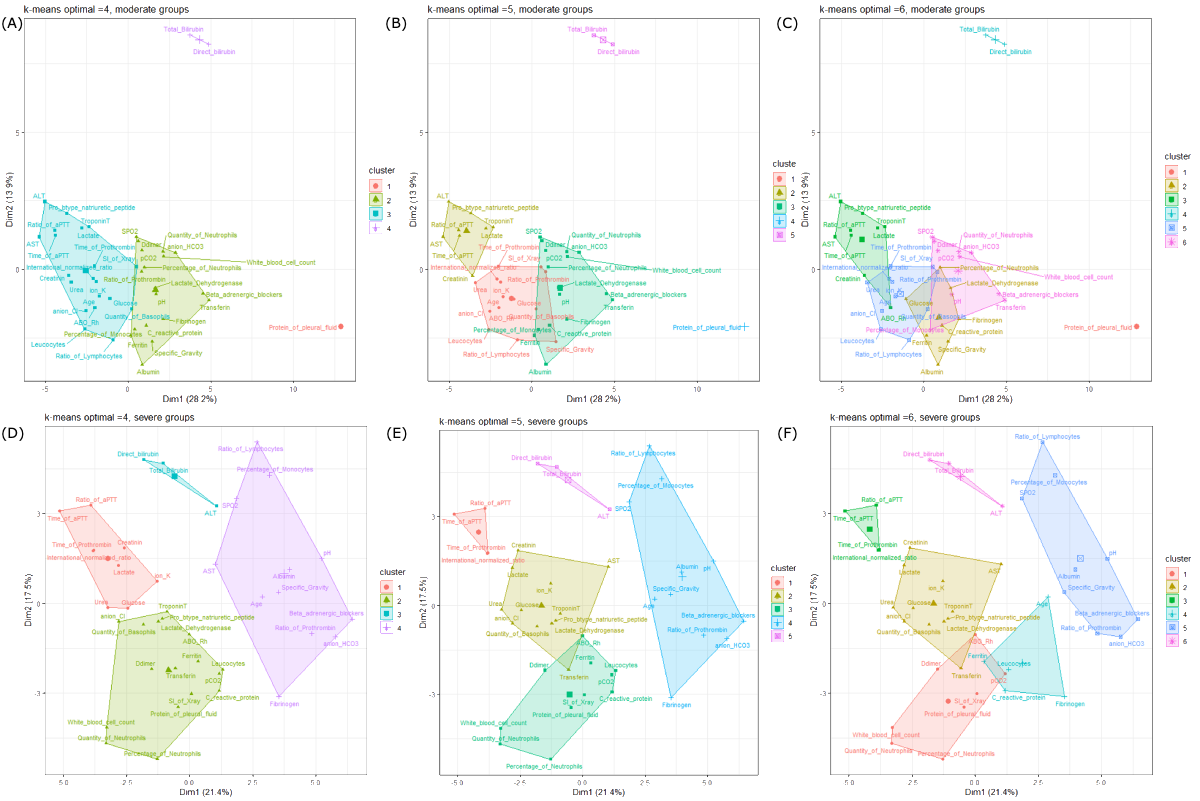
Principal component analysis cluster plot of the moderate group (A-C) and the severe group (D-F) in 2-dimensional space (dim1 and dim2). These dimensions can be interpreted as principal components (PCs). In the moderate group, the PCs accounted for 42.1% (17.5% [dim1] + 17.5% [dim2]) of the total variation. In the severe group, the PCs accounted for 38.9% (21.4% [dim1] + 17.5% [dim2]) of the variation in the data set.

The results of k-means clustering in the 2 linkage models “complete” and “average” with 20 different index values showed that with our unfiltered data (66 parameters), the parameters could be separated into 3 to 5 clusters; however, the results of k-means did not indicate coherence between the groups. The Ratkowsky index proposed 3 or 4 clusters in all groups, the mild group, and the moderate group, but 13 clusters in the severe group (Multimedia Appendix 16). Thus, it is important to further assess the filtered data after eliminating unbalance parameters and selecting the ones with significant *P* values for relative risk and RR (Multimedia Appendix 17). We selected the results proposed by the tau and Ratkowsky methods, which proposed from 2 to 6 clusters as optimal. The PCA cluster plots in Figures 6 and 7 show that 4 is the best number to appropriately distinguish the clusters and avoid overlap. The different distributions of the preclinical factors were fitted with the correlation results. The PCA plot arranged the variables in 2-dimensional space (dim1 and dim2). These dimensions can be interpreted as principal components (PCs), which are the largest symbols located in the middle of every cluster. PCs are linear combinations of variables. The percentage of dim indicates how much of the total variation the PC accounts. In all 3 groups, the mild group, the moderate group, and the severe group, the PCs accounted for 42.0%, 40.1%, 41.2%, and 38.9%, respectively, of the total variation. COVID-19 severity was associated with age, glucose in the blood, CRP, LDH, scoring index of chest x-ray, quantity and percentage of neutrophils, WBC count, fibrinogen, specific gravity, and protein in the pleural fluid. Further assessing every severity group, the group of factors may include pH, D-dimer, and troponin T in the mild group and transferrin, albumin, FiO_2_, anion HCO_3_, beta-adrenergic blockers, pCO_2_, and glucose in the moderate group, or may be split into 2 different clusters in the severe group. This confirms the inversion of the correlation direction of certain factors via their distances (eg, albumin/direct bilirubin and ratio of monocytes (%), pCO_2_ and pH, scoring index of chest x-ray and protein in the pleural fluid, ALT and leucocytes, albumin/fibrinogen/LDH and protein in the pleural fluid, D-dimer and protein in the pleural fluid, international normalized ratio and quantity of leucocytes, and protein in the pleural fluid and lactate) (Figures 6 and 7).

**Table 2:** Correlation between cophenetic distances and the original distance data.

Linkage mode	All	Mild disease	Moderate disease	Severe disease
ward.D	0.3358179	0.5429852	0.4238019	0.5572885
ward.D2	0.3827203	0.5236514	0.477412	0.6228183
single	0.8181252	0.7414184	0.7740626	0.6131261
complete	0.6922355	0.6047242	0.6705475	0.5952852
average	0.8602272	0.8020538	0.8437752	0.7472646
mcquitty	0.7541622	0.7962945	0.8373778	0.7280575
median	0.7829029	0.6169923	0.7621790	0.4760746
centroid	0.8118045	0.7619601	0.7430496	0.6353752

### Network Building

The clustering evaluation procedure has to tackle challenging problems, such as the quality of clusters, the degree to which a clustering scheme fits a specific data set, and the optimal number of clusters in partitioning. Taking all the above results and using the MDS layout, we continued to look at the influence of COVID-19 severity on other factors and how their link was disturbed by other neighbors independently using the HCA and k-means process. Based on the *r* values, the data to be analyzed is a collection of *M* objects (in our study, it will be the number of preclinical factors in each imputation) on which a distance function defines the entries of the dissimilarity matrix. The distance between factors in the network showed how well they connect and which other factors could disturb their link (Multimedia Appendix 18). The network is the last step, which provides the relation of the selected factors in a 3-dimensional map (Figure 6).

We found that COVID-19 severity was directly linked to age, scoring index of chest x-ray, percentage of neutrophils, albumin, CRP, and ratio of lymphocytes. Age, scoring index of chest x-ray, percentage of neutrophils, and CRP showed a strong positive correlation with COVID-19 severity. Moreover, their exact RRs based on cutoff_50_ values significantly differed from the pooled RR. In the mild-moderate group, age of 77.56 years was a significant cutoff_50_, with an RR of 4.19 (95% CI 3.58-4.95; *P*<.001). This RR was higher than the pooled RR of 2.78 (*P*<.001) by about 1.41, and the probability of mild patients becoming moderate patients increased 3 times. In this group, the *r* value between age and severity was 0.44 (*P*<.001) (Table 1). Percentage of neutrophils was noted in all of the groups. In the mild-moderate group, the cutoff_50_ value was 84.80% (*P*<.001), with an RR of 3.18 (95% CI 2.73-3.70; *P*<.001), which was higher than the pooled RR of about 0.40, and the probability of mild patients becoming moderate patients increased 2 times. In the moderate-severe group, the cutoff_50_ value was 87.74% (*P*<.001), with an RR of 3.32 (95% CI 2.6480-4.1529; *P*<.001), which was higher than the pooled RR of about 2.04, and the probability of moderate patients becoming severe patients increased 2.3 times. In the moderate-severe group, the cutoff_50_ value of the number of neutrophils was 11.77 G/L (*P*<.001), with an RR of 3.15 (95% CI 2.6153-3.8025; *P*<.001), which was higher than the pooled RR of about 1.25, and the probability of moderate patients becoming severe patients increased 2.15 times. The scoring index of chest x-ray (Brixia) was noted in all of the groups. In the mild-moderate group, the cutoff_50_ value was 5.53 (*P*<.001), with an RR of 3.29 (95% CI 2.76-3.92; *P*<.001), which was higher than the pooled RR of about 0.51, and the probability of mild patients becoming moderate patients increased 4.5 times. In the moderate-severe group, the cutoff_50_ value was 10.51 (*P*<.001), with an RR of 3.03 (95% CI 2.4023-3.8314; *P*<.001), which was higher than the pooled RR of 1.28 (95% CI 1.22-1.33; z=11.23; *P*<.001), and the probability of moderate patients becoming severe patients increased 2 times. In the mild-moderate group, CRP had a cutoff_50_ value of 7.46 mg/dL (*P*<.001), with an RR of 3.4 (95% CI 2.91-3.97; *P*<.001), which was higher than the pooled RR of about 0.62, and the probability of mild patients becoming moderate patients increased 2.4 times. In the moderate-severe group, albumin had a cutoff_50_ value of 29.73 g/L (*P*<.001), with an RR of 0.46 (95% CI 0.3650-0.5752; *P*<.001), which was lower than the pooled RR of about 0.82, and the probability of moderate patients becoming severe patients decreased 0.54 times. In the moderate-severe group, ratio of lymphocytes had a cutoff_50_ value of 6.32% (*P*<.001), with an RR of 0.34 (95% CI 0.2743-0.4210; *P*<.001), which was lower than the pooled RR of about 0.94, and the probability of moderate patients becoming severe patients decreased 0.66 times.

The significant inversion of correlations among the severity groups is important. On assessing the results in more detail, important factors, which have been mentioned above, showed weak and medium links with other factors besides severity (Table 1; Multimedia Appendix 1 and Multimedia Appendix 4). The most important shift was from the following 2 pairs: (1) transferrin and anion Cl, and (2) ALT and leucocytes. The pair of ALT and leucocytes showed an important negative correlation (*r*=−1; *P*<.001) in the mild group and a significant positive correlation in the moderate group (*r*=1; *P*<.001). The mean values of ALT in the mild and moderate groups were 81.92 (IQR 19.00-132.00) U/L and 193.51 (IQR 100.00-213.00) U/L, respectively. The mean values of leucocytes in the mild and moderate groups were 242.96 (IQR 100.00-500.00) cells/µL and 201.39 (IQR 15.00-500.00) cells/µL, respectively. There was a significant increase in ALT (*P*<.001). The pair of transferrin and anion Cl showed an important positive correlation (*r*=1; *P*<.001) in the mild group and a significant negative correlation in the moderate group (*r*=0.59; *P*<.001). The mean values of transferrin in the mild and moderate groups were 312.67 (IQR 255.50-354.00) mg/dL and 159.87 (IQR 141.00-181.50) mg/dL, respectively. The mean values of anion Cl in the mild and moderate groups were 98.4 (IQR 97.00-101.00) mmol/L and 95.84 (IQR 93.00-99.00) mmol/L, respectively.

**Figure 8:**
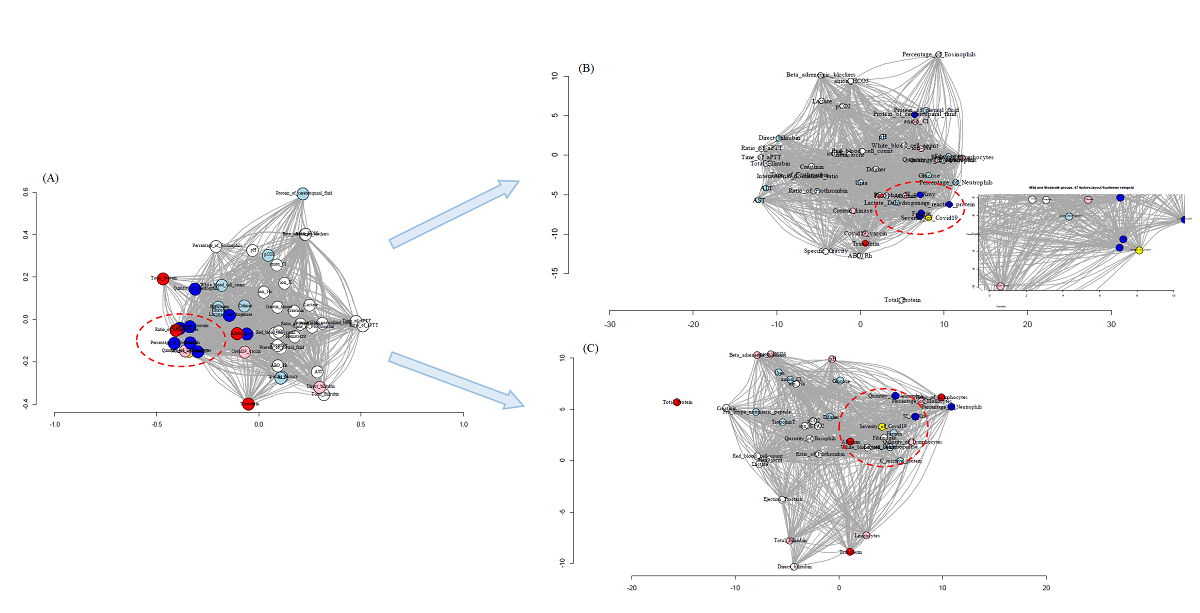
Network plot for the mild-moderate group and moderate-severe group (2 dimensions). (A) All groups (47 factors, multidimensional scaling [MDS] layout). (B) Mild-moderate group (47 factors, MDS layout). (C) Moderate-severe group (37 factors, MDS layout). Yellow indicates COVID-19 severity; red indicates that the factor has a strong negative correlation with COVID-19 severity (−0.4>*r*>−1); blue indicates that the factor has a strong positive correlation with COVID-19 severity (0.4<*r*<1); pink indicates that the factor has a moderately negative correlation with COVID-19 severity (−0.20>*r*>−0.40); light blue indicates that the factor has a moderately positive correlation with COVID-19 severity (0.20<*r*<0.40); and grey indicates that the factor has a weak correlation with COVID-19 severity (−0.20<*r*<0.20). Further details are provided in Multimedia Appendices 19 and 20.

The network map and PCA plot showed that the closest neighbors of COVID-19 severity were ferritin and age, and CRP, scoring index of chest x-ray, albumin, and LDH were the next closest neighbors of these 3 factors. These findings were consistent with the correlation results, in which these factors showed a weak correlation with other subclinical indices (Multimedia Appendix 4) and a strong correlation with COVID-19 severity (Table 1; Figure 8; Multimedia Appendix 18, Multimedia Appendix 19 and Multimedia Appendix 20).

We found different factors close to COVID-19 severity in the moderate-severe group. These were ferritin, fibrinogen, albumin, quantity of lymphocytes, scoring index of chest x-ray, WBC count, LDH, and quantity of neutrophils. In the whole group map, we found the following factors: age, scoring index of chest x-ray, number of lymphocytes, percentage of neutrophils, ferritin, and CRP. The distances between age/ferritin/quantity of lymphocytes and COVID-19 severity were shorter than the distances between age/ferritin/quantity of lymphocytes and other factors, which indicated a direct link among these 4 factors. Scoring index of chest x-ray, number of lymphocytes, percentage of neutrophils, and CRP also had close links to severity.

## Discussion

### Overview

Our ML study included 2173 patients (1587 mild and asymptomatic patients, 377 moderate patients, and 209 severe patients). Imputation was run first in 2 correlation tests. Then, the relative risk and RR were used to eliminate unbalanced parameters and select the most remarkable parameters. HCA and k-means were independently employed to classify the parameters according to their *r* values. Finally, network analysis provided a 3-dimensional view of the results. COVID-19 severity was significantly correlated with age (mild-moderate group: RR 4.19, 95% CI 3.58-4.95; *P*<.001), scoring index of chest x-ray (mild-moderate group: RR 3.29, 95% CI 2.76-3.92; *P*<.001; moderate-severe group: RR 3.03, 95% CI 2.4023-3.8314; *P*<.001), percentage of neutrophils (mild-moderate group: RR 3.18, 95% CI 2.73-3.70; *P*<.001; moderate-severe group: RR 3.32, 95% CI 2.6480-4.1529; *P*<.001), quantity of neutrophils (moderate-severe group: RR 3.15, 95% CI 2.6153-3.8025; *P*<.001), albumin (moderate-severe group: RR 0.46, 95% CI 0.3650-0.5752; *P*<.001), CRP (mild-moderate group: RR 3.4, 95% CI 2.91-3.97; *P*<.001), and ratio of lymphocytes (moderate-severe group: RR 0.34, 95% CI 0.2743-0.4210; *P*<.001). The significant inversion of correlations among the severity groups is important. ALT and leucocytes showed a significant negative correlation in the mild group (*r*=−1; *P*<.001) and a significant positive correlation in the moderate group (*r*=1; *P*<.001). Moreover, transferrin and anion Cl showed a significant positive correlation in the mild group (*r*=1; *P*<.001) and a significant negative correlation in the moderate group (*r*=−0.59; *P*<.001). The clustering and network analysis visualized that in the mild-moderate group, the closest neighbors of COVID-19 severity were ferritin and age. CRP, scoring index of chest x-ray, albumin, and LDH were the next closest neighbors of these 3 factors. In the moderate-severe group, the closest neighbors of COVID-19 severity were ferritin, fibrinogen, albumin, quantity of lymphocytes, scoring index of chest x-ray, WBC count, LDH, and quantity of neutrophils.

### COVID-19 Severity is Directly Associated With Age, Scoring Index of Chest X-Ray, Percentage of Neutrophils, Albumin, CRP, and Ratio of Lymphocytes

Age, scoring index of chest x-ray, percentage of neutrophils, and CRP showed a strong positive correlation with COVID-19 severity. Moreover, their exact RRs based on cutoff_50_ values significantly differed from the pooled RR.

There are many views on the age-related difference in COVID-19 severity. The explanation for the marked age gradient is likely multifactorial. The proposed mechanisms that relate specifically to the pathogenesis of SARS-CoV-2 seem more likely to be critical than those that would also apply to other viral infections for which a similar age gradient is not seen. Differences in innate, adaptive, and heterologous immunity and differences in endothelial and clotting function are potential mechanisms to explain the observed age gradient in COVID-19. Following exposure to SARS-CoV-2, immunologic factors in children are essential for preventing or controlling the virus after infection. Age-related differences in endothelial and clotting function are associated with an increased risk of COVID-19 complications in elderly patients, which can lead to higher mortality [13]. This is consistent with our finding that 77.56 years was a significant cutoff_50_ for age (RR 4.19, 95% CI 3.58-4.95; *P*<.001) and that age and severity were correlated (*r*=0.44; *P*<.001) in the mild-moderate group. This RR was higher than the pooled RR of 2.78 (*P*<.001) by about 1.41, and the probability of mild patients becoming moderate patients increased 3 times. We did not see an important shift from mild/moderate disease to severe disease associated with age, and several other parameters may have stronger influences on this outcome.

Neutrophils are a typical type of WBC produced by the bone marrow and stimulate phagocytosis to attack bacteria. Under normal conditions, neutrophils have a value of around 2-8 G/L, equivalent to 43%-76% of the total leukocytes. Neutrophil totals are used in clinical practice to assess the body’s ability to fight infections, particularly bacterial infections. Patients are considered to have neutropenia when the absolute blood count is below 2000/μL and an increased risk of disease when the count is below 1000/μL. Neutrophil decrease may be due to decreased production or increased peripheral destruction. Percentage of neutrophils was identified in all of the study groups. The risk of mild patients becoming moderate patients increased 2 times when the percentage of neutrophils was greater than 84.80% (*P*<.001), and the risk of moderate patients becoming severe patients increased about 2 times when the percentage of neutrophils was greater than 87.74% (*P*<.0001) and their quantity was more than 11.77 G/L (*P*<.001). Having a high percentage of neutrophils in the blood is called neutrophilia, and this is a sign of infection (most likely bacterial). The increase of the neutrophil percentage in our results is consistent with the aggravation of the infection status from mild to moderate and from moderate to severe.

Substantial evidence has accumulated since the beginning of the COVID-19 pandemic that neutrophils play an essential role in the pathophysiology of COVID-19, particularly in those with severe disease courses [14]. SARS-CoV-2 engages the inflammasome in monocytes and macrophages, leading to a cytokine storm. Neutrophils, the most abundant leukocytes, release neutrophil extracellular traps (NETs), which have been implicated in the pathogenesis of COVID-19. A recent study by Aymonnier et al [15] showed that activation of the NLRP3 inflammasome is essential for NET release in sterile inflammation. Their clinical study showed that in COVID-19 infection, ASC specks are formed in neutrophils, and the percentage of neutrophils showing a speck is similar to that of mononuclear cells. Given their vast presence in the blood and lungs compared with other types of leukocytes, we can conclude that neutrophils could be the primary producers of specks. These findings support the possibility that neutrophils and monocytes contribute to the cytokine storm through the inflammasome, and ASC speck detection in vivo could indicate innate immune system activation. Bohnacker et al [16] showed in a recent study that monocyte-derived macrophages (MDMs) cause the inflammatory response to SARS-CoV-2, and they are a significant source of eicosanoids in airway inflammation. RNA sequencing analysis identified 163 differentially expressed genes in MDMs differentiated from monocytes in seropositive individuals 3 to 5 months after infection compared with MDMs in seronegative subjects. Post–COVID-19 MDMs showed higher expression of proinflammatory chemokines (CCL2, CCL8, and CCL7), driving neutrophil recruitment, including in COVID-19.

Integration of imaging and clinical parameters could improve the stratification of COVID-19 patients on emergency department admission. Monaco et al [17] showed that according to receiver operating characteristic curve analysis, the optimal cutoff values for Brixia score and patient age were 8 points and 71 years, respectively. Similarly, Hoang et al [18] showed that in univariate analysis, age, vaccination status, previous disease, NEWS2, SpO_2_, the Brixia score, and the Total Severity Score were signiﬁcant predictors of mortality (*P*<.05). In multivariate analysis, age, SpO_2_, the Brixia score, and previous disease were independent predictors of mortality and hospitalization. In our cohort, the scoring index of chest x-ray (Brixia) was used, and in all groups, it appeared as a potential marker. Our results are consistent with the findings of Monaco et al and provide more information regarding COVID-19 severity. The risk of the disease status changing from mild to moderate increased 4.5 times when the scoring index was more than 5.53 (*P*<.001), and the risk of the disease status changing from moderate to severe increased 2 times when the scoring index was more than 10.51 (*P*<.001).

CRP is produced by the liver, and it binds to polysaccharide C of pneumococcus. CRP is one of the proteins released into the bloodstream in response to inflammation and is considered an early marker of infection and inflammation. The CRP test is a quantitative blood test for CRP, which measures the overall level of inflammation in the body. Normal CRP levels are deficient (<0.5 mg/dL). At the onset of inflammation, CRP in the blood rises rapidly within 6 to 8 hours. As the inflammation or tissue damage resolves, CRP levels drop. Thus, although CRP is a nonspecific indicator of inflammation, it is a valuable marker for monitoring disease severity. In pooled data, when CRP was higher than 20.69 mg/dL (*P*<.001), the probability of the disease status changing from mild/moderate to severe was 50% (Multimedia Appendix 13). CRP was one of the most important factors in the mild-moderate group. Its cutoff_50_ value was 7.46 mg/dL (*P*<.001), with an RR of 3.4 (95% CI 2.91-3.97; *P*<.001), which was higher than the pooled RR of about 0.62, and the probability of mild patients becoming moderate patients increased 2.4 times. As CRP was located in the same cluster as age (mild/moderate group; Figures 5 and 6), they showed a positive correlation. Similar findings were noted in a recent study by Faucheux et al [19]. There were 2 cohorts of COVID-19 hematological patients from France and Brazil during the prevaccination period, and 2 profiles of patients were identified. One profile included young patients with few comorbidities and low CRP, D-dimer, LDH, and creatinine levels, and the other profile included older patients with several comorbidities and high levels of the 4 biological markers. The profiles were strongly associated with survival (*P*<.001), even after adjusting for age (*P*<.001). The plasma CRP level was positively correlated with COVID-19 severity, and a higher CRP level was associated with a longer inpatient duration. For the first time, the plasma CRP level was demonstrated to assist in discerning patients with moderate to severe COVID-19 pneumonia from those with mild conditions. This suggests that CRP testing may be useful as an earlier indicator for severe illness and may help physicians to stratify patients for intensive care unit transfer [20].

Albumin is an important protein, accounting for a large part (58%-74%) of the total protein in the body. It is produced in the liver at about 10.5 g/day, with the main functions of preventing water from going out of blood vessels, maintaining colloidal osmotic pressure at a stable level, and binding and transporting fatty acids, steroid hormones, vitamins, bilirubin, and drugs to every organ in the body. In our study, albumin and ratio of lymphocytes showed a strong negative correlation with COVID-19 severity. The exact RR based on the cutoff_50_ value showed a large difference from the pooled RR. When albumin was higher than 29.73 g/L (*P*<.001), the probability of moderate patients becoming severe patients decreased 0.54 times. Hypoalbuminemia is associated with critical illness and mortality across numerous clinical settings. The pathophysiology behind hypoalbuminemia in the disease state is thought to be secondary to increased capillary permeability, decreased protein synthesis, the half-life of serum albumin, serum albumin total mass, increased volume of distribution, and increased expression of vascular endothelial growth factor [21]. The lymphocyte index reflects the number of lymphocytes present in the body. This is essential in helping doctors understand the patient’s condition and detect abnormalities early, so they can diagnose and treat them promptly. In normal people, the number of WBCs usually has a very high range, with an average value of 4 to 10 G/L and a percentage in the blood of 17%-48%. The risk of moderate patients becoming severe patients decreased 0.66 times when the ratio of lymphocytes was lower than 6.32% (*P*<.001) in the moderate-severe group (RR 0.34, 95% CI 0.2743-0.4210; *P*<.001). Yamasaki et al [22] showed in their study in 2020 that lymphocyte counts approximately 6 days after disease onset were significantly lower in the severe pneumonia group compared to both the nonsevere pneumonia group and the nonpneumonia group (*P*=.02 and *P*=.002, respectively). The severe pneumonia group had a low mean lymphocyte count at 659 (SD 318.9) cells/mm^3^. Patients in the severe pneumonia group were significantly older than those in the nonsevere pneumonia group (*P*=.008) but not significantly different from those in the nonpneumonia group [22].

### Significant Inversion of the Correlation Among Severity Groups

On assessing the results in more detail, the important factors, which have been mentioned above, showed weak and medium correlations with other factors besides severity (Multimedia Appendix 1, Multimedia Appendix 4, and Multimedia Appendix 14). The most relevant shift was noted for the following 2 pairs: (1) transferrin and anion Cl, and (2) ALT and leucocytes.

The pair of ALT and leucocytes showed a significant negative correlation (*r*=−1; *P*<.001) in the mild group and a significant positive correlation in the moderate group (*r*=1; *P*<.001). The mean values of ALT in the mild and moderate groups were 81.92 (IQR 19.00-132.00) U/L and 193.51 (IQR 100.00-213.00) U/L, respectively. The mean values of leucocytes in the mild and moderate groups were 242.96 (IQR 100.00-500.00) cells/µL and 201.39 (IQR 15.00-500.00) cells/µL, respectively. There was a significant increase in ALT (*P*<.001). ALT might have a stronger influence on the inversion than leucocytes. The complete blood count (CBC) is the most common examination used to monitor overall health in clinical practice. It is unclear whether there is a relationship between CBC indexes and ALT and AST. Aminotransferases include AST and ALT, and they are markers of hepatocellular injury. They are also correlated with obesity, with the normal reference range being higher in those with higher BMI [23]. Leukocytes can be induced to express tissue factors and release proinflammatory and procoagulant molecules (granular enzymes, cytokines, and damage-associated molecular patterns). These intermediaries can influence all aspects of thrombus formation, including platelet activation, adhesion, and activation of the intrinsic and extrinsic coagulation pathways [24].

The pair of transferrin and anion Cl showed a significant positive correlation (*r*=1; *P*<.001) in the mild group and a significant negative correlation in the moderate group (*r*=0.59; *P*<.001). The mean values of transferrin in the mild and moderate groups were 312.67 (IQR 255.50-354.00) mg/dL and 159.87 (IQR 141.00-181.50) mg/dL, respectively. The mean values of anion Cl in the mild and moderate groups were 98.4 (IQR 97.00-101.00) mmol/L and 95.84 (IQR 93.00-99.00) mmol/L, respectively. The major function of human transferrin is to deliver iron from the bloodstream to actively dividing cells. Upon iron release, the protein changes its conformation from “closed” to “open.” Studies in vitro indicated that iron release from transferrin is very complex and involves many factors, including pH, the chelator used, the anion effect, temperature, receptor binding, and intralobe interactions. The nature of the dual effect of chloride is as follows: the anion effect on iron release is closely related to the strength of anion binding to the apoprotein. The negative effect seems to originate from competition between chloride and the chelate for an anion-binding site near the metal center [25]. Lower transferrin is associated with more anion Cl release in plasma.

### Limitations

This multidimensional analysis study showed possible correlations between several elements and COVID-19 severity to provide clinical reference markers for surveillance and diagnostic management and to enhance the prevention of severe outcomes. This is the main advantage of the clustering concept. However, this concept has some limitations. HCA and k-means are the unsupervised clustering methods that we used to group the data. These approaches use a random and unlabeled data set, and their results are displayed in a dendrogram and PCA plot. When clustering multidimensional data, before using HCA or k-means, it is very important to clean and prepare the data set, because these approaches cannot be run with missing or noisy data. As HCA proposed only 3 solutions for classification, we needed to combine and validate the data with k-means, which did not propose a dendrogram, but it provided several possibilities of the optimal cluster number to build a PCA cluster plot and determine the PC position. Since our data had different types of information, it was difficult to compute the distance matrix in HCA and k-means. Data settings at the beginning could help us work with both qualitative and numerical data at the same time.

### Conclusion

The network map and PCA plot showed that the closest neighbors of COVID-19 severity were ferritin and age. CRP, scoring index of chest x-ray, albumin, and LDH were the next closest neighbors of these 3 factors. These findings were consistent with the correlation results, in which these factors showed a weak correlation with other subclinical indices (Multimedia Appendix 4 and Multimedia Appendix 14). In the PCA plot of the entire cohort, the distances between age/ferritin/quantity of lymphocytes and COVID-19 severity were shorter than the distances between age/ferritin/quantity of lymphocytes and other factors, which indicated a direct link among these factors. Scoring index of chest x-ray, number of lymphocytes, percentage of neutrophils, and CRP also had a close link to severity. In the moderate-severe group, albumin, WBC count, fibrinogen, and LDH had a close link to severity, in addition to ferritin, quantity of lymphocytes, scoring index of chest x-ray, and quantity of neutrophils.

ML techniques are attracting substantial interest from medical researchers and clinicians following visible successes in multiple predictive tasks [9,26]. Classifying data is a common task in ML. If data points need to be split into 2 groups, the ability to decide in which group a new data point will be included will provide clarity on preclinical factors, and this might depend on not only the severity of patients but also other factors. Our data sets may serve as a basis for further studies where the data can be combined reasonably with data from similar studies to understand the impact of different factors on the outcomes of COVID-19.

## Appendix

Multimedia Appendix 1 Patient medical history (2173 COVID-19 patients).

Multimedia Appendix 2 Pearson correlation test (*r* values) in the 2173 COVID-19 patients.

Multimedia Appendix 3 Pearson correlation test (*P* values) in the 2173 COVID-19 patients.

Multimedia Appendix 4 Sixty-seven subclinical factors from 2173 COVID-19 patients according to disease severity.

Multimedia Appendix 5 Inverse correlations in the mild, moderate, and severe groups.

Multimedia Appendix 6 Pearson correlation test (*r* values) in the mild group.

Multimedia Appendix 7 Pearson correlation test (*P* values) in the mild group.

Multimedia Appendix 8 Pearson correlation test (*r* values) in the moderate group.

Multimedia Appendix 9 Pearson correlation test (*P* values) in the moderate group.

Multimedia Appendix 10 Pearson correlation test (*r* values) in the severe group.

Multimedia Appendix 11 Pearson correlation test (*P* values) in the severe group.

Multimedia Appendix 12 *P* values from the pairwise *t* test comparing the correlation (*r*) of each factor with other factors among the mild, moderate, and severe groups.

Multimedia Appendix 13 Predicted values linked with a 50:50 probability of COVID-19 patients changing from a mild/moderate status to a severe status.

Multimedia Appendix 14 Most important factors in the mild-moderate group.

Multimedia Appendix 15 Most important factors in the moderate-severe group.

Multimedia Appendix 16 Results of clustering imputation involving 66 parameters (linkage model: complete).

Multimedia Appendix 17 Results of clustering imputation involving 43 parameters for all groups (selected from the second correlation test) and 41 parameters for each group (selected from the relative risk and risk ratio *P* value; linkage model: complete and average).

Multimedia Appendix 18 Network plot for mild-moderate 3 dimensions. Node in yellow color: severity of COVID-19. Node in red color: factors have strong negative correlation with COVID-19 severity (-0.4>R>-1). Node in blue color: factors have strong positive correlation with COVID-19 severity (0.4<R<1). Node in pink color: factors have moderate negative correlation with COVID-19 severity (-0.20>R>-0.40). Node in color light-blue: factors have moderate positive correlation with COVID-19 severity (0.20<R<0.40). Node in grey color: factors have weak correlation with COVID-19 severity (-0.20<R<0.20). (See more in Multimedia Appendix 9).

Multimedia Appendix 19 Network plot for moderate-severe group, 3 dimensions. Node in yellow color: severity of COVID-19. Node in red color: factors have strong negative correlation with COVID-19 severity (-0.4>R>-1). Node in blue color: factors have strong positive correlation with COVID-19 severity (0.4<R<1). Node in pink color: factors have moderate negative correlation with COVID-19 severity (-0.20>R>-0.40). Node in color light-blue: factors have moderate positive correlation with COVID-19 severity (0.20<R<0.40). Node in grey color: factors have weak correlation with COVID-19 severity (-0.20<R<0.20). (See more in Multimedia Appendix 9).

Multimedia Appendix 20 Network plot for all groups (3 dimensions).

## Abbreviations

ALT: alanine transaminase
AST: aspartate transaminase
CBC: complete blood count
CRP: C-reactive protein
HCA: hierarchical cluster analysis
LDH: lactate dehydrogenase
MDM: monocyte-derived macrophage
MDS: multidimensional scaling
ML: machine learning
NET: neutrophil extracellular trap
PC: principal component
PCA: principal component analysis
pro-BNP: pro–B-type natriuretic peptide
RR: risk ratio
WBC: white blood cell

## Data Availability

High-resolution images of the figures included in the paper can be obtained on request from the corresponding author.
