# Supplementary material for: Multidimensional Machine Learning for Assessing Parameters Associated With COVID-19 in Vietnam: Validation Study
Source: JMIR Form Res. 2023 Feb 16;7:e42895. doi: 10.2196/42895 (PMC9937111; doi:10.2196/42895)
Supplement: Multimedia Appendix 1 [file formative_v7i1e42895_app1.pdf]

Multimedia Appendix 1. Patient medical history (2173 COVID-19 patients).

**Diagnostic Report**	**Number of patients**	**Number patients in the mild and non symptom group**	**Number patients in the moderate group**	**Number patients in the severe group**
Diabetes	315	155	107	53
Lung related disease	195	64	57	74
Shock	72	8	1	63
Cardio related disease	559	278	176	105
Hepatic related disease	93	58	25	10
Brain infarction related disease	87	35	36	16
Neural related disease	38	11	18	9
Renal related disease	168	82	42	44
Cancer	102	61	19	22
Bone cartilage related disease	56	29	20	7
Digestive systems related disease	107	145	18	7
Immune related disease	30	23	4	3
**ICD-10-CM (http://icd.kcb.vn/)**				**Number of patients**
W18 (Slipping, tripping, stumbling and falls)				1
W01 (Slipping, tripping, stumbling and falls)				1
Z80_Z89 (Family history of malignant neoplasm of digestive organs)				1
Z70_Z76 (persons encountering health services for examination and investigation)				1
Z40_Z54 (Persons encountering health services for specific procedures and health care)				9
V52 (Accidents)				1
T66_T78 (Other and unspecified effects of external causes)				1
T15_T19 (Effects of foreign body entering through natural orifice)				1
T00_T07 (Injuries involving multiple body regions)				1
U04 (Severe acute respiratory syndrome, unspecified)				1
U07.1 (COVID-19, virus identified)				927

U07.2 (COVID-19, virus not identified)	4
Z00_Z13 (Persons encountering health services for examination and investigation)	1
S80_S89 (Injuries to the knee and lower leg)	2
S70_S79 (Injuries to the hip and thigh)	1
S50_S59 (Injuries to the elbow and forearm)	2
S40_S49 (Injuries to the shoulder and upper arm)	1
S30_S39 (Injuries to the abdomen, lower back, lumbar spine and pelvis)	3
S20_S29 (Injuries to the thorax)	1
S00_S09 (Injuries to the head)	1
R57.2 (Septic shock)	1
R57.1 (Hypovolaemic shock)	1
Q60_Q64 (Congenital malformations of the urinary system)	1
Q38_Q45 (Other congenital malformations of the digestive system)	1
Q30_Q34 (Congenital malformations of the respiratory system)	1
N80_N89 (Noninflammatory disorders of female genital tract)	1
N40_N51 (Diseases of male genital organs)	1
N30_N39 (Other diseases of urinary system)	1
N17_N19 (Renal failure)	1
M80_M94 (Osteopathies and chondropathies)	3
M60_M79 (Soft tissue disorders)	2
M40_M54 (Dorsopathies)	11
M00_M25 (Arthropathies)	4
K90_K93 (Other diseases of the digestive system)	1
K80_K87 (Disorders of gallbladder, biliary tract and pancreas)	2
K70_K77 (Diseases of liver)	1
K65_K67 (Diseases of peritoneum)	1
K55_K64 (Other diseases of intestines)	8
K50_K52 (Noninfective enteritis and colitis)	1
K40_K46 (Hernia)	1
K35_K38 (Diseases of appendix)	1
K20_K31 (Diseases of oesophagus, stomach and duodenum)	1
I10_I15 (Hypertensive diseases)	7
J90_J94 (Other diseases of pleura)	1

J80_J84 (Other respiratory diseases principally affecting the interstitium)	1
J09_J18 (Influenza and pneumonia)	1
G90_G99 (Other disorders of the nervous system)	1
G80_G83 (Cerebral palsy and other paralytic syndromes)	1
F40_F48 (Neurotic, stress-related and somatoform disorders)	1
F00_F09 (Organic, including symptomatic, mental disorders)	1
E70_E90 (Metabolic disorders)	1
E20_E35 (Disorders of other endocrine glands)	1
E10_E14 (Diabetes mellitus)	4
D65_D69 (Coagulation defects, purpura and other hemorrhagic conditions)	2
D37_D48 (Neoplasms of uncertain or unknown behavior)	1
D10_D36 (Benign neoplasms)	1
D00_D09 (In situ neoplasms)	1
C00_C97 (Neoplasms)	4
B35_B49 (Mycoses)	1
B25_B34 (Other viral diseases)	11
B15_B19 (Viral hepatitis)	1
A30_A49 (Other bacterial diseases)	1
