# Supplementary material for: Multidimensional Machine Learning for Assessing Parameters Associated With COVID-19 in Vietnam: Validation Study
Source: JMIR Form Res. 2023 Feb 16;7:e42895. doi: 10.2196/42895 (PMC9937111; doi:10.2196/42895)
Supplement: Multimedia Appendix 4 [file formative_v7i1e42895_app4.pdf]

Appendix table : 67 subclinical factors from 2173 Covid19 patients, 3 groups of patients: Severe, Moderate and Mild. (cont)

		MILD (N=1587 patients)							MODERATE(N=377 patients)							SEVERE(N=209 patients)							P-value of one way ANOVA		
		Min.	1stQu.	Median	Mean	3rdQu	Max.	no information	Min.	1stQu.	Median	Mean	3rdQu	Max.	no information	Min.	1stQu.	Median	Mean	3rdQu	Max.	no information	Mild vs Moderate	Moderate vs Severe	Mild vs Severe
1	SPO_2 (%)	7	97	98	97.2	98	100	608	80	95	96	95.77	98	100	64	35	87	94	89.84	97	100	16	7.2×10^{-7}	$<2E\times10^{-16}$	$<2E\times10^{-16}$
2	Age (years old)	1	27	43	44.72	62	108	10	8	59	72	68.48	83	99	3	17	62	73	70.20	83	103	2	2.8×10^{-69}	2.7×10^{-1}	1.6×10^{-50}
3	Gender(1= Male, 2 = Female)	1	1	2	1.52	2	2	11	1	1	2	1.51	2	2	3	1	1	1	1.45	2	2	2	5.5×10^{-1}	1.9×10^{-1}	5.1×10^{-2}
4	Severity of Covid19 (1= Mild, 2=Moderate, 3=Severe)	1	1	1	1	1	1	1	2	2	2	2	2	2	2	3	3	3	3	3	3	3	NA	NA	NA
5	Covid19 vaccine (0 = no. 1=1 boost, 2= 2 boosts, 3= 3 boosts)	0	0	2	1.58	3	3	971	0	0	0	0.90	2	3	278	0	0	0	0.85	2	3	155	2.6×10^{-5}	8.0×10^{-1}	2.8×10^{-4}
6	Urobilinogen (μmol/L)	3	3	3	3	3	3	1074	3	3	3	3	3	3	363	1	3	3	2.90	3	3	161	NA	NA	7.1×10^{-1}
7	Unrinary Creatinine (umol/L)	676	3761.50	6860	5963	9061.50	9456	1089	NA	NA	NA	NaN	NA	NA	377	7818	7818	7818	7818	7818	7818	208	NA	NA	NA
8	Urea (mmol/L)	1.30	3.80	4.60	5.31	5.80	34.70	316	1.60	4.50	5.70	6.94	7.88	39.60	23	2	6.50	9.40	11.34	13.95	45.80	2	5.3×10^{-13}	1.0×10^{-18}	4.9×10^{-63}
9	Urinary Glucose (mmol/L)	3	5.88	15.50	26.69	56	56	1085	5.50	5.50	6	21.04	35	56	365	3	5.50	14	20.48	28	56	153	6.0×10^{-1}	9.3×10^{-1}	4.1×10^{-1}
10	Cetone (mmol/L)	1.50	1.50	1.50	6	8.25	15	1090	0.50	1.25	1.50	3.38	2.38	15	369	0.50	0.50	1.50	3.13	1.50	15	179	5.1×10^{-1}	8.9×10^{-1}	3.2×10^{-1}
11	Leucocytes (LEU/uL)	15	100	125	242.69	500	500	1080	15	15	85	201.39	500	500	359	1	25	70	101.1	100	500	161	6.0×10^{-1}	3.3×10^{-2}	6.4×10^{-3}
12	pH	5.50	7	7.41	7.14	7.44	8	1001	5	7.39	7.42	7.34	7.45	8	134	5	7.09	7.35	6.91	7.43	7.50	15	1.6×10^{-4}	1.1×10^{-11}	2.0×10^{-2}
13	Specific Gravity	1005	1012	1015	1016.62	1020	1033	1048	1007	1013	1019	1019.65	1026	1042	340	1006	1016	1021	1021	1026	1047	98	7.9×10^{-2}	2.8×10^{-1}	8.6×10^{-4}
14	SI of ^{Xra}y	1	2	3	3.55	4	15	705	1	4	7	6.98	9	15	74	2	10	12	11.08	13	16	24	1.5×10^{-49}	1.0×10^{-34}	2.5×10^{-34}
15	Ejection Fraction	32	45.75	57	53.70	60.75	69	1083	17	41.50	62	55.79	67	78	358	30	44	53	57.29	75	85	188	7.2×10^{-1}	7.7×10^{-1}	5.5×10^{-1}
16	Lactate (mmol/L)	1	1.40	1.90	2.39	2.40	17.80	1028	0.80	1.60	2	2.41	2.60	20	140	0.60	1.60	2.30	2.75	3	20	18	9.4×10^{-1}	8.4×10^{-2}	2.6×10^{-1}
17	Fibrinogen (g/L)	0.74	2.76	3.26	3.35	3.81	8.45	391	0.87	3.12	3.88	4.08	4.72	9.63	63	0.64	3.32	4.34	4.46	5.67	9.46	21	1.6×10^{-20}	8.6×10^{-3}	2.3×10^{-29}
18	Percentage of Monocytes (%)	0	5.40	7.40	7.77	9.60	60	252	0	3.20	5.30	6.02	7.90	21.40	9	0	NULL	NULL	0	NULL	NULL	0	3.5×10^{-13}	2.2×10^{-16}	9.4×10^{-46}
19	Quantity of Monocytes (G/L)	0.02	0.33	0.45	0.50	0.61	1.66	265	0	0.23	0.36	0.46	0.61	3.06	11	0	NULL	NULL	0	NULL	NULL	0	4.0×10^{-2}	2.6×10^{-1}	4.6×10^{-4}
20	Mean Corpuscular volume (fL)	63.40	87.90	91.10	90.43	94.40	119.7	253	59.10	88.48	91.70	90.78	95.20	126.60	9	65.80	89.50	92.70	91.93	96.43	121.90	3	4.3×10^{-1}	1.0×10^{-1}	NA
21	Mean Corpuscular hemoglobin (pg/cell)	18	28	30	38.95	31	344	253	18	29	30	32.63	31	329	9	0	NULL	NULL	0	NULL	NULL	0	3.1×10^{-2}	4.8×10^{-1}	3.1×10^{-2}
22	Mean Corpuscular hemoglobin concentration (g/dL)	29	317	325	323.42	334	360	253	30	317.8	324	323.23	332	362	9	0	NULL	NULL	0	NULL	NULL	0	8.9×10^{-1}	3.3×10^{-1}	3.1×10^{-1}

Appendix table : 67 subclinical factors from 2173 Covid19 patients, 3 groups of patients: Severe, Moderate and Mild. (cont)

		MILD (N=1587 patients)							MODERATE(N=377 patients)							SEVERE(N=209 patients)							p value of ANOVA		
		Min.	1stQu.	Median	Mean	3rdQu	Max.	no information	Min.	1stQu.	Median	Mean	3rdQu	Max.	no information	Min.	1stQu.	Median	Mean	3rdQu	Max.	no information	Mild vs Moderate	Moderate vs Severe	Mild vs Severe
23	Quantity of Lymphocytes (G/L)	0.09	0.89	1.25	1.36	1.71	7.43	266	0.09	0.58	0.89	1	1.29	3.80	10	0.11	0.32	0.56	0.70	0.82	3.49	1	4.0×10^{-19}	1.6×10^{-9}	5.5×10^{-36}
24	Percentage of Eosinophils (%)	0.20	0.50	0.70	0.88	1.08	1.90	1089	0	0.08	0.10	1.03	1.05	3.90	373	0	0	0	0	0	0	208	8.8×10^{-1}	6.6×10^{-1}	3.6×10^{-1}
25	Quantity of Basophils (G/L)	0	0.02	0.04	0.05	0.06	1.03	258	0	0.02	0.03	0.05	0.05	0.80	9	0	0.02	0.04	0.07	0.07	2.60	1	5.4×10^{-1}	2.5×10^{-2}	3.7×10^{-3}
26	Percentage of Basophils (%)	0	0.40	0.60	0.75	0.90	10.90	267	0	0.30	0.40	0.60	0.70	6.90	11	0	0.20	0.30	0.48	0.60	3.30	1	5.1×10^{-4}	1.1×10^{-2}	1.7×10^{-7}
27	Red blood cell count	2.19	4.32	4.71	4.70	5.12	6.92	268	2.25	4.07	4.53	4.51	5	6.73	11	1.44	3.84	4.40	4.35	4.90	6.79	1	1.7×10^{-5}	1.2×10^{-2}	2.9×10^{-10}
28	White blood cell count (G/L)	0.34	4.92	6.37	6.77	7.93	29.51	263	0.15	5.46	7.38	9.03	9.72	306	12	2.14	8.28	12.50	13.22	16.91	40.43	4	1.6×10^{-4}	6.6×10^{-4}	3.5×10^{-78}
29	Hemoglobin (T/L)	100	129	139	139.21	150	191	286	100	121.8	133	133.93	146	190	25	0	NULL	NULL	0	NULL	NULL	0	5.9×10^{-7}	1.1×10^{-1}	5.9×10^{-9}
30	Ratio of Lymphocytes (%)	1	14	21	22.32	28.60	77.90	268	1	7.68	12.05	14.81	19.53	66	9	0.60	2.55	5.40	6.66	8.70	34	2	1.1×10^{-26}	6.9×10^{-25}	1.3×10^{-71}
31	Quantity of Neutrophils (G/L)	0	2.80	4.10	4.63	5.60	28.70	264	0.20	3.80	5.55	6.62	8.23	55.50	13	1.80	7.20	11.25	11.79	15.53	38.10	1	2.9×10^{-18}	3.5×10^{-24}	2.5×10^{-03}
32	Percentage of Neutrophils (%)	0.80	57.10	66.30	65.34	75.60	97.20	256	2.60	67.53	79.55	75.99	86.10	95.90	11	47.70	84.58	89.50	87.97	93.23	98.20	1	4.4×10^{-31}	3.6×10^{-29}	1.5×10^{-86}
33	Hematocrit (L/L)	0.19	0.39	0.43	0.62	0.46	48	255	0.17	0.37	0.41	0.41	0.45	0.63	9	0.14	0.36	0.41	0.40	0.45	0.59	1	1.5×10^{-1}	7.0×10^{-2}	2.6×10^{-1}
34	Beta adrenergic blockers (mmol/L)	33.10	42.10	43.85	43.53	45.63	51.50	1029	26.90	42.40	44.70	44.48	46.53	56.10	153	3	39.15	42.70	41.83	44.95	56.80	2	6.7×10^{-2}	7.0×10^{-8}	3.5×10^{-2}
35	anion HCO3 (mmol/L)	6.20	20.05	22.10	21.37	23.10	30.10	1026	13.70	20.90	22.95	22.95	24.90	36.10	139	6.10	18.68	21.50	21.18	23.63	42.20	9	6.4×10^{-4}	9.1×10^{-6}	7.7×10^{-1}
36	anion Cl (mmol/L)	74	97	99	98.34	101	107	263	73	93	96	95.84	99	126	8	76	95	99	99.45	104	132	1	1.4×10^{16}	3.7×10^{-9}	3.6×10^{-3}
37	ion K (mmol/L)	2.50	3.60	3.80	3.86	4.10	6.40	264	2.20	3.60	3.90	3.94	4.30	6.10	10	2.30	3.70	4	4.58	4.40	100	9	3.9×10^{-3}	7.4×10^{-2}	2.5×10^{-3}
38	ion Na (mmol/L)	114	134	137	135.80	138	145	264	110	130	133	133.30	137	165	9	106	132	135	135.7	138	169	6	2.6×10^{-17}	3.0×10^{-5}	8.4×10^{-1}
39	pCO2 (mmHg)	16.10	30.75	34.10	33.64	37.55	47	1026	23.10	31.95	35.30	36.46	39.10	79.60	138	15.80	30.88	36.90	40.71	45.43	122.70	5	4.0×10^{-3}	9.5×10^{-5}	1.4×10^{-4}
40	FiO_2 (Fraction of inspired oxygen)	0.21	0.21	0.21	0.21	0.21	0.21	1026	0	0.21	0.21	0.21	0.21	0.21	138	0.21	0.21	0.21	0.22	0.21	1	4	5.8×10^{-1}	1.0×10^{-1}	4.4×10^{-1}
41	INR (International normalized ratio)	0.89	1.17	1.35	1.45	1.60	4.36	967	0.97	1.21	1.36	1.50	1.59	4.85	260	0.93	1.27	1.42	1.56	1.64	7.37	29	4.2×10^{-1}	3.8×10^{-1}	9.1×10^{-2}
42	Time of Prothrombin (s)	11.90	14.90	16.60	17.48	19	41.80	966	12.80	15.30	16.8	18	19.05	44.90	259	12.50	15.80	17.30	18.44	19.33	62.10	29	3.4×10^{-1}	4.6×10^{-1}	8.3×10^{-2}
43	Ratio of Prothrombin (%)	17	52	65	65.22	78.50	120	966	14.90	51	62.5	66.79	74	633	259	12	51	60	60.63	70	112	29	7.6×10^{-1}	1.6×10^{-1}	2.5×10^{-2}
44	D-dimer (ng/mL)	100	194	310	777.29	639.50	27100	303	20	329.75	585	1223.35	1157.50	26700	23	100	903.50	1550	4149	3960	36400	10	5.5×10^{-4}	2.0×10^{13}	3.1×10^{-35}

Appendix table : 67 subclinical factors from 2173 Covid19 patients, 3 groups of patients: Severe, Moderate and Mild.

		MILD (N=1587 patients)							MODERATE(N=377 patients)							SEVERE(N=209 patients)							p value of ANOVA		
		Min.	1stQu.	Median	Mean	3rdQu	Max.	no informatio	Min.	1stQu.	Median	Mean	3rdQu	Max.	no informatio	Min.	1stQu.	Median	Mean	3rdQu	Max.	no informatio	Mild vs Moderate	Moderate vs Severe	Mild vs Severe
45	Time of aPTT (s)	24.30	29.85	32.20	34.06	35.20	100	999	21.80	28.83	32.1	34.90	37.53	189.70	283	20	29.75	34.10	40.15	42.25	123.30	30	6.8×10^{-1}	2.5×10^{-2}	3.7×10^{-3}
46	Ratio of aPTT (Disease/control)	0.90	1.10	1.10	1.17	1.20	1.90	999	0.80	1.03	1.10	1.25	1.30	6.70	283	0.80	1.08	1.20	1.32	1.50	4.70	33	2.6×10^{-1}	2.8×10^{-1}	4.0×10^{-3}
47	Total Protein	NA	NA	NA	NA	NA	NA	1093	54.80	54.80	54.8	54.80	54.80	54.80	376	48.50	48.50	48.50	48.50	48.50	48.50	208	NA	NA	NA
48	Protein of pleural fluid (g/L)	11.40	11.50	23.60	22.18	23.60	40.80	1088	21.50	26.28	31.1	31.05	35.83	40.60	375	2	19.70	27.50	25.53	32.78	40.70	201	4.3×10^{-1}	5.8×10^{-1}	6.3×10^{-1}
49	Protein of cerebrospinal fluid	0.33	0.41	0.49	0.52	0.61	0.73	1090	0.24	0.99	1.73	1.73	2.48	3.23	374	0.81	0.81	0.81	0.81	0.81	0.81	208	2.3×10^{-1}	6.4×10^{-1}	3.3×10^{-1}
50	Glucose of cerebrospinal fluid	3.72	3.72	3.72	3.72	3.72	3.72	1092	2.41	4.34	5.16	4.86	5.69	6.72	373	6.17	6.17	6.17	6.17	6.17	6.17	208	6.1×10^{-1}	5.6×10^{-1}	NA
51	Glucose (mmol/L)	2.90	5.30	6.10	6.89	7.40	29.80	340	3.50	5.95	7.30	9.34	10.30	46.70	42	0.60	6.90	10.15	12.74	15.03	89.50	45	4.1×10^{-21}	1.5×10^{-6}	3.1×10^{-39}
52	Albumin (g/L)	18	31.58	36.50	35.92	40.90	49.30	937	18.10	29.20	32.9	32.72	35.70	45.70	180	7.20	24.10	27.80	27.68	31	41.10	32	3.8×10^{-7}	1.1×10^{-18}	1.4×10^{-28}
53	Transferrin (mg/dL)	230	255.50	281	312.67	354	427	1090	66	141	155	159.87	181.5	265	362	64	89	112	118.6	141	239	178	7.9×10^{-4}	3.5×10^{-3}	5.8×10^{-8}
54	Pro Calcitonin (ng/mL)	0.05	0.24	2.75	16.83	10.72	100	1085	0.03	0.10	0.58	41.66	8.20	418	361	0.06	0.50	1.63	29.03	7	616	114	5.3×10^{-1}	6.3×10^{-1}	7.2×10^{-1}
55	Ferritin (ng/mL)	7.42	144.20	338.17	466.19	626.21	2000	304	9.87	427.7	919.7	992.66	1676	2000	39	97.80	1088	1676	1422	1720.75	2000	25	2.2×10^{-52}	1.3×10^{-14}	2.9×10^{-11}
56	C reactive protein (mg/dL)	0.07	0.30	0.70	1.96	1.94	21.50	333	0.10	1.58	4.20	6.47	8.90	41.10	37	0.10	3.60	8.70	10.66	16.10	38.60	56	6.4×10^{-44}	1.4×10^{-8}	4.2×10^{-83}
57	Pro b-type natriuretic peptide (pro-BNP, pg/ml)	5	35.01	93.20	385.55	428.85	4983	977	5	102.1	348.9	1330.09	913.7	35000	195	12.70	413	926	3836	2891	35000	64	1.0×10^{-2}	9.4×10^{-5}	9.2×10^{-7}
58	Troponin-T (ng/L)	3	7	10.70	21.45	18	423.7	956	3.70	10.35	15.9	36.88	28.13	760.70	165	4.30	17.25	37.05	146.4	90.25	3253	43	3.3×10^{-2}	3.1×10^{-5}	9.9×10^{-5}
59	Creatinine (µmol/L)	19	56	69	76.88	87	954	252	18	58	74	84.11	93	852	13	24	62	85	107.8	124	608	12	2.8×10^{-2}	1.7×10^{-4}	8.2×10^{-13}
60	Phosphor (mmol/L)	0.70	0.90	1	1.17	1.28	2.50	1079	0.40	0.80	1	1.03	1.20	2.30	332	0.30	0.80	1	1.07	1.23	3.40	77	2.2×10^{-1}	6.6×10^{-1}	4.3×10^{-1}
61	Creatin kinase (U/L 37°C)	48	105	184	1217.23	792	9764	1080	32	84	102	345.93	197	2800	362	20	102.25	249	716.7	739.25	5294	129	2.3×10^{-1}	1.9×10^{-1}	2.2×10^{-1}
62	Lactate Dehydrogenase (U/L 37°C)	93	177	202	232.12	244	5548	348	144	229	311	352.40	409	3146	36	145	386.50	540.5	579.2	665.25	3518	29	9.4×10^{-17}	9.8×10^{-19}	1.3×10^{-57}
63	Total Bilirubin (µmol/L)	5.10	7.10	8.40	26.27	9.70	136.8	1086	4	5	7.30	43.90	9.20	194	372	4	8.50	15.50	20.61	23.40	61.20	188	6.5×10^{-1}	2.3×10^{-1}	6.5×10^{-1}
64	Direct bilirubin (µmol/L)	3.20	3.40	5.30	31.38	14.40	130.6	1088	2.70	2.85	3	62.80	92.85	182.70	374	2.40	4.20	6.80	11.28	11.45	50.30	186	5.8×10^{-1}	1.5×10^{-2}	1.1×10^{-1}
65	AST (U/L 37°C)	14	26	44	108.25	130.50	1570	1014	20	101.75	128	243.91	257.50	3109	323	16	104	126	232	196	2376	122	1.3×10^{-2}	8.6×10^{-1}	8.5×10^{-3}
66	ALT (U/L 37°C)	11	19	43	81.92	132	441	1008	15	100	125	193.51	213	1815	332	19	102	124	335.4	190	4049	150	8.2×10^{-4}	1.9×10^{-1}	1.0×10^{-3}
67	ABO Rh (1 = O/+, 2 = A/+, 3=B/+, 4=AB/+)	N(1)=4	N(2)=7	N(3)=2	N(4)=15			1065	N(1)=7	N(2)=5	N(3)=1	N(4)=9			355	N(1)=20	N(2)=18	N(3)=4	N(4)=23			144	6.8×10^{-1}	2.5×10^{-2}	3.7×10^{-3}

Appendix table : 67 subclinical factors from 2173 Covid19 patients, 3 groups of patients: Severe, Moderate and Mild.
