# Supplementary material for: Multidimensional Machine Learning for Assessing Parameters Associated With COVID-19 in Vietnam: Validation Study
Source: JMIR Form Res. 2023 Feb 16;7:e42895. doi: 10.2196/42895 (PMC9937111; doi:10.2196/42895)
Supplement: Multimedia Appendix 5 [file formative_v7i1e42895_app5.pdf]

Multimedia Appendix 5. Inverse correlations in the mild, moderate, and severe groups.

1st factor	2nd factor	R scores MILD	P values MILD	R scores MODERATE	P values MODERATE	R scores SEVERE	P values SEVERE	Inverse correlation
Urea	anion Cl	-0.20	1.4×10^{-08}	0.29	4.2×10^{-08}	0.32	1.9×10^{-06}	- in mild to + in moderate/ severe
Leucocytes	ALT	-1.00	4.5×10^{-308}	1.00	0.0×10^{-00}	-0.34	2.1×10^{-01}	- in mild to + in moderate
Ratio of Lymphocytes	Creatinine	-0.08	1.7×10^{-02}	-0.02	6.4×10^{-01}	0.24	6.1×10^{-04}	- in mild to + in severe
Beta adrenergic blockers	ALT	-0.11	7.3×10^{-01}	-0.47	6.1×10^{-03}	0.28	2.8×10^{-02}	- in moderate to + in severe
anion Cl	Urea	-0.20	1.4×10^{-08}	0.29	4.2×10^{-08}	0.32	1.9×10^{-06}	- in mild to + in moderate/ severe
anion Cl	D-dimer	-0.10	7.8×10^{-03}	0.07	2.1×10^{-01}	0.21	2.8×10^{-03}	- in mild to + in severe
anion Cl	Transferrin	1.00	0.0×10^{-00}	-0.59	2.0×10^{-02}	0.05	7.8×10^{-01}	+ in mild to - in moderate
D-dimer	Protein of pleural fluid	-0.70	1.8×10^{-01}	1.00	4.5×10^{-308}	0.82	1.2×10^{-02}	- in mild to + in moderate/ severe
Protein of pleural fluid	Lactate	0.00	4.5×10^{-308}	-1.00	4.5×10^{-308}	0.43	3.3×10^{-01}	- in moderate to + in severe
Protein of pleural fluid	Fibrinogen	1.00	4.1×10^{-02}	1.00	4.5×10^{-308}	-0.37	4.1×10^{-01}	+ in mild to - in severe
Albumin	Direct bilirubin	1.00	4.5×10^{-308}	-1.00	4.5×10^{-308}	-0.30	2.3×10^{-01}	+ in mild to - in moderate/ severe
Direct bilirubin	ALT	-0.94	2.2×10^{-01}	0.00	4.5×10^{-308}	0.76	4.8×10^{-02}	- in mild to + in severe
ALT	Total Bilirubin	-0.61	3.8×10^{-01}	0.00	4.5×10^{-308}	0.85	3.0×10^{-02}	- in mild to + in severe

SPO2	Transferrin	1.00	$4.5\times10_{-308}$	0.46	1.0×10^{-01}	-0.46	8.9×10^{-03}	+ in mild/moderate to - in severe
Age	Glucose	0.22	7.7×10^{-10}	0.04	5.2×10^{-01}	-0.20	9.2×10^{-03}	+ in mild to - in severe

The color value of the cells is proportional to the strength of the associations, ranging from red (negative correlations) to blue (positive correlations).
