# Supplementary material for: Multidimensional Machine Learning for Assessing Parameters Associated With COVID-19 in Vietnam: Validation Study
Source: JMIR Form Res. 2023 Feb 16;7:e42895. doi: 10.2196/42895 (PMC9937111; doi:10.2196/42895)
Supplement: Multimedia Appendix 12 [file formative_v7i1e42895_app12.pdf]

Multimedia Appendix 12. *P* values from the pairwise *t* test comparing the correlation (*r*) of each factor with other factors among the mild, moderate, and severe groups.

	SEVERE vs MODERATE	SEVERE vs MILD	MODERATE vs MILD
**Albumin**	5.0×10^{-02}	8.0×10^{-02}	9.9×10^{-05}
**ALT**	2.7×10^{-01}	4.1×10^{-02}	6.2×10^{-02}
**anion Cl**	5.6×10^{-03}	3.9×10^{-03}	6.8×10^{-02}
**anion HCO3**	1.1×10^{-03}	9.0×10^{-05}	6.8×10^{-05}
**Beta-adrenergic blockers**	1.2×10^{-02}	5.0×10^{-04}	4.5×10^{-04}
**C reactive protein**	1.2×10^{-01}	1.8×10^{-03}	4.4×10^{-04}
**Creatinine**	9.2×10^{-04}	3.2×10^{-04}	7.7×10^{-03}
**D-dimer**	2.6×10^{-04}	6.7×10^{-01}	6.5×10^{-01}
**Ejection Fraction**	9.7×10^{-02}	2.7×10^{-02}	8.4×10^{-02}
**Ferritin**	3.0×10^{-03}	9.8×10^{-05}	1.4×10^{-02}
**Fibrinogen**	6.1×10^{-01}	1.1×10^{-01}	3.2×10^{-03}
**FiO2**	6.0×10^{-01}	1.1×10^{-01}	3.2×10^{-03}
**Glucose**	6.0×10^{-01}	1.1×10^{-01}	6.5×10^{-01}
**ion K**	8.4×10^{-02}	5.3×10^{-02}	3.3×10^{-06}
**Lactate**	1.5×10^{-01}	8.5×10^{-02}	2.4×10^{-01}
**Lactate Dehydrogenase**	4.0×10^{-01}	3.8×10^{-03}	3.3×10^{-02}
**Percentage of Neutrophils**	4.0×10^{-01}	3.8×10^{-03}	2.3×10^{-07}
**pH**	3.2×10^{-03}	9.9×10^{-03}	3.5×10^{-05}

**Pro b-type natriuretic peptide**	3.1×10^{-04}	3.7×10^{-01}	6.6×10^{-01}
**Protein of pleural fluid**	9.8×10^{-01}	9.6×10^{-01}	5.4×10^{-01}
**Quantity of Basophils**	2.1×10^{-04}	6.2×10^{-02}	3.7×10^{-01}
**Quantity of Neutrophils**	7.6×10^{-03}	1.8×10^{-01}	3.2×10^{-04}
**Ratio of Lymphocytes**	1.5×10^{-01}	2.0×10^{-01}	8.6×10^{-08}
**SI of $^{X\text{-}r}$ay**	5.3×10^{-01}	3.6×10^{-05}	7.0×10^{-01}
**White blood cell count**	3.6×10^{-03}	3.8×10^{-01}	1.8×10^{-02}
**SPO2**	1.6×10^{-03}	2.6×10^{-02}	5.1×10^{-09}
