# Supplementary material for: Multidimensional Machine Learning for Assessing Parameters Associated With COVID-19 in Vietnam: Validation Study
Source: JMIR Form Res. 2023 Feb 16;7:e42895. doi: 10.2196/42895 (PMC9937111; doi:10.2196/42895)
Supplement: Multimedia Appendix 13 [file formative_v7i1e42895_app13.pdf]

Multimedia Appendix 13. Predicted values linked with a 50:50 probability of COVID-19 patients changing from a mild/moderate status to a severe status.

	CUTOFF50 50PERCENT	ESTIMATE	STD-ERROR	Z-VALUE	P-R(>|Z|)	N PATIENTS IN UPPER	N PATIENTS IN LOWER	N SEVERE PATIENT IN UPPER	N SEVERE PATIENT IN LOWER	N OTHER PATIENTS IN UPPER	N OTHER PATIENTS IN LOWER	NORMAL VALUE		
												Female	Male	units
**SPO_2**	87.80	-0.200	0.02160	-9.240	2.4×10^{-20}	1112	74	144	49	968	25	95-100	95-100	%
**UREA**	15.87	0.207	0.018	11.500	1.3×10^{-30}	82	1463	36	171	46	1292	2.76 - 8.07	2.76 - 8.07	mmol/L
**LEUCOCYTES**	273.69	-0.004	0.001	-2.670	7.6×10^{-3}	16	80	5	43	11	37	<10	<10	LEU/uL
**PH**	6.52	-0.880	0.152	-5.780	7.3×10^{-9}	606	84	150	44	456	40	4.8 - 7.4	4.8 - 7.4	
**SPECIFIC GRAVITY**	1013.98	0.054	0.020	2.770	5.6×10^{-3}	178	54	90	21	88	33	1.005 - 1.030	1.005 - 1.030	
**SI OF ^{XRA}Y**	11.03	0.446	0.032	14.100	5.2×10^{-45}	211	815	103	82	108	733			
**LACTATE**	8.33	0.079	0.046	1.740	8.1×10^{-2}	10	639	6	185	4	454	0.6 - 1.4	0.6 - 1.4	mmol/L
**FIBRINOGEN**	7.75	0.447	0.056	7.960	1.7×10^{-15}	23	1357	6	182	17	1175	2.00 - 4.00	2.00 - 4.00	g/L
**PERCENTAGE OF MONOCYTES**	1.21	-0.453	0.037	-12.100	9.7×10^{-34}	1594	39	200	8	1394	31	0.00 - 0.80	0.00 - 0.80	%
**QUANTITY OF BASOPHILS**	0.76	2.470	1.060	2.320	2.0×10^{-2}	3	1623	1	207	2	1416	0.00 - 0.10	0.00 - 0.10	G/L
**WHITE BLOOD CELL COUNT**	21.18	0.145	0.015	9.590	8.8×10^{-22}	48	1565	28	177	20	1388	4.00 - 10.00	4.00 - 10.00	G/L
**RATIO OF LYMPHOCYTES**	3.99	-0.239	0.018	-13.100	2.6×10^{-39}	1450	165	124	83	1326	82	20 - 45	20 - 45	%
**QUANTITY OF NEUTROPHILS**	14.27	0.259	0.019	13.700	9.4×10^{-43}	113	1503	66	142	47	1361	1.8 -7.5	1.8 -7.5	G/L
**PERCENTAGE OF NEUTROPHILS**	90.67	0.180	0.013	13.600	2.4×10^{-42}	186	1440	87	121	99	1319	45 - 75	45 - 75	%
**BETA ADRENERGIC BLOCKERS**	40.22	-0.112	0.022	-5.100	3.3×10^{-7}	528	121	143	64	385	57	45 - 52	45 - 52	mmol/l
**ANION HCO3**	17.04	-0.087	0.024	-3.690	2.2×10^{-4}	601	62	168	32	433	30	21.00 - 29.50	21.00 - 29.50	mmol/L
**ANION CL**	127.08	0.061	0.014	4.490	7.0×10^{-6}	3	1620	2	206	1	1414	98.0 - 107.0	98.0 - 107.0	mmol/L
**ION K**	6.12	0.843	0.151	5.600	2.1×10^{-8}	6	1599	4	196	2	1403	3.40 - 4.50	3.40 - 4.50	mmol/L
**$_PCO_2$**	47.10	0.044	0.010	4.540	5.6×10^{-6}	73	597	46	158	27	439	35.00 - 45.00	35.00 - 45.00	mmHg
**FIO_2**	0.21	1340.0	60500	0.022	9.8×10^{-1}	2	667	2	203	0	464			
**INTERNATIONAL NORMALIZED RATIO**	2.56	0.285	0.185	1.540	1.2×10^{-1}	18	494	6	174	12	320	0.8 - 1.2	0.8 - 1.2	
**TIME OF PROTHROMBIN**	27.87	0.031	0.021	1.480	1.3×10^{-1}	20	494	7	173	13	321			
**RATIO OF PROTHROMBIN**	30.98	-0.010	0.006	-1.760	7.7×10^{-2}	492	22	173	7	319	15	70.00 - 140.0	70.00 - 140.0	%
**DDIMER**	8676.77	0.00025	0.00003	7.990	1.3×10^{-15}	51	1498	26	173	25	1325	<500.00	<500.00	ng/mL
**TIME OF APTT**	38.63	0.025	0.008	3.000	2.6×10^{-3}	98	350	50	129	48	221			
**RATIO OF APTT**	1.36	0.603	0.288	2.090	3.6×10^{-2}	100	345	51	125	49	220	0.8-1.2	0.8-1.2	times
**TOTAL PROTEIN**	51.65	-7.480	17800	-0.0004	1.00	1	2	0	1	1	1	64 - 83	64 - 83	g/L
**PROTEIN OF PLEURAL FLUID**	4.32	0.006	0.046	0.139	8.8×10^{-1}	15	1	7	1	8	0	0.15 - 0.45	0.15 - 0.45	g/L
**GLUCOSE**	23.60	0.133	0.015	8.850	8.4×10^{-19}	41	1400	15	149	26	1251	4.56 - 6.38	4.11 - 5.89	mmol/L

**ALBUMIN**	27.29	-0.192	0.020	-9.460	$3.0×10^{-21}$	501	159	98	79	403	80	35 - 52	35 - 52	g/L
**TRANSFERIN**	166.85	-0.024	0.008	-2.920	$3.4×10^{-3}$	12	49	3	28	9	21	200 - 360	200 - 360	mg/dL
**FERRITIN**	1887.07	0.002	0.0002	13.900	$1.2×10^{-43}$	110	1396	38	146	72	1250	10 - 291	30 - 400	ng/ mL
**C REACTIVE PROTEIN**	20.69	0.135	0.012	11.100	$1.2×10^{-28}$	58	1385	24	129	34	1256	<0.50	<0.50	mg/dL
**PRO BTYPE NATRIURETIC PEPTIDE**	6569.28	0.00015	0.00004	4.110	$3.9×10^{-5}$	44	521	25	120	19	401	<125	<125	pg/mL
**TROPONIN-T**	167.28	0.007	0.001	4.730	$2.2×10^{-6}$	44	608	27	139	17	469	<14.0	<14.0	ng/L
**CREATININ**	382.15	0.006	0.001	4.840	$1.3×10^{-6}$	15	1602	3	194	12	1408	44 - 80	62 - 106	umol/L
**LACTATE DEHYDROGENASE**	659.69	0.006	0.0005	12.300	$9.1×10^{-35}$	85	1374	48	132	37	1242	135 - 214	135 - 225	U/L
**TOTAL BILIRUBIN**	95.27	-0.008	0.009	-0.867	$3.8×10^{-1}$	2	40	0	21	2	19	<15	<24	µmol/l
**DIRECT BILIRUBIN**	72.14	-0.021	0.014	-1.540	$1.2×10^{-1}$	2	37	0	23	2	14	≤3.4	≤3.4	µmol/l
**AST**	886.48	0.001	0.0004	1.380	$1.6×10^{-1}$	6	258	3	84	3	174	≤32	≤40	U/I
**ALT**	588.41	0.002	0.001	2.440	$1.4×10^{-2}$	15	216	7	52	8	164	≤33	≤41	U/I
