# Supplementary material for: Multidimensional Machine Learning for Assessing Parameters Associated With COVID-19 in Vietnam: Validation Study
Source: JMIR Form Res. 2023 Feb 16;7:e42895. doi: 10.2196/42895 (PMC9937111; doi:10.2196/42895)
Supplement: Multimedia Appendix 14 [file formative_v7i1e42895_app14.pdf]

Multimedia Appendix 14. Most important factors in the mild-moderate group.

**MILD vs MODERATE** RR=2.78 [2.66;2.90] (z=45.29, *P*<.000001)	**Percentage of Neutrophils**	**Protein of pleural fluid**	**C reactive protein (CRP)**	**Ferritin**	**Age**	**SI of Xray**	**Protein of cerebrospinal fluid**
cutoff50 mild to moderate	84.8	38.98	7.46	1194.7	77.56	5.53	0.92
Estimate	6.05E-02	7.36E-02	2.02E-01	1.70E-03	5.24E-02	4.26E-01	1.88E+00
Std-Error	5.55E-03	8.14E-02	1.84E-02	1.29E-04	3.47E-03	3.63E-02	1.81E+00
Z-value	1.09E+01	9.05E-01	1.10E+01	1.32E+01	1.51E+01	1.17E+01	1.04E+00
P	$1.13×10^{-27}$	$3.66×10^{-01}$	$4.40×10^{-28}$	$1.25×10^{-39}$	$1.92×10^{-51}$	$9.09×10^{-32}$	$2.97×10^{-01}$
N patients in upper of cutoff50	171	2	148	204	238	247	2
N patients in lower of cutoff50	1201	13	1141	1082	1610	490	5
N moderate patients upper of cutoff50	114	1	104	137	143	189	2
N moderate patients lower of cutoff50	252	1	236	201	231	114	1
N other patient in upper of cutoff50	57	1	44	67	95	58	0
N other patient in lower of cutoff50	949	12	905	881	1379	376	4
**RR**	**3.18**	**6.5**	**3.4**	**3.62**	**4.19**	**3.29**	**3.67**
95%-CI	[2.73-3.7]	[0.63-67.35]	[2.91-3.97]	[3.09-4.23]	[3.58-4.9]	[2.77-3.92]	[0.94-14.35]
%W(common)	5.1	0	4.4	5.2	4.8	6.2	0.1
test statistic	5.14E+01	1.88E-01	2.07E+02	1.92E-29	2.66E+02	2.21E+01	2.72E-01
df	1	1	1	1	1	1	1
p value 1s	$3.74×10^{-13}$	$3.33 ×10^{-01}$	$3.85 ×10^{-47}$	$5.00 ×10^{-01}$	$4.52 ×10^{-60}$	$1.27 ×10^{-06}$	$3.01 ×10^{-01}$
p value 2s	$7.48 ×10^{-13}$	$6.65 ×10^{-01}$	$7.70 ×10^{-47}$	1.00 ×10+00	$9.03 ×10^{-60}$	$2.54 ×10^{-06}$	$6.02 ×10^{-01}$
R score with Severity of Covid19	0.33	0.36	0.4	0.43	0.44	0.52	0.57

P(pearson) with Severity of Covid19	4.51×10^{-308}	4.30E-01	4.51×10^{-308}	4.51×10^{-308}	4.51×10^{-308}	4.51×10^{-308}	2.35E-01

The Meta analytical method, such as: the Mantel-Haenszel method, the restricted maximum-likelihood estimator for tauX2, the Q-profile method for confidence interval of tauX2 and tau, the continuity correction of 0.5 in studies with zero cell frequencies, we found in the **Mild – Moderate group for 47 factors**: RR=2.78 [2.66;2.90] (z=45.29, *P*<.000001), tauX2=0.2635 [0.2043;0.9629], tau=0.5133 [0.4520;0.9813], IX2=91.6% [89.7%;93.1%], H=3.45 [3.11;63.82]. Test of heterogeneity: Q=546.33, d.f. = 46, *P* <0.0001. The Outcome + : moderate, the Outcome -: mild**.**
