# Supplementary material for: Multidimensional Machine Learning for Assessing Parameters Associated With COVID-19 in Vietnam: Validation Study
Source: JMIR Form Res. 2023 Feb 16;7:e42895. doi: 10.2196/42895 (PMC9937111; doi:10.2196/42895)
Supplement: Multimedia Appendix 15 [file formative_v7i1e42895_app15.pdf]

Multimedia Appendix 15. Most important factors in the moderate-severe group.

**MODERATE vs SEVERE**<br>RR=1.28 [1.22;1.33]<br>(z=11.23, *P*<.0001)	**Quantity of Neutrophils**	**Ratio of Lymphocytes**	**Transferrin**	**Albumin**	**Percentage of Neutrophils**	**Direct bilirubin**	**SI of X-ray**
cutoff50 moderate to severe	11.77	6.32	170.73	29.73	87.74	108.97	10.51
Estimate	1.79E-01	-1.71E-01	-2.16E-02	-1.87E-01	1.31E-01	-2.45E-02	3.53E-01
Std-Error	2.04E-02	1.88E-02	8.48E-03	2.44E-02	1.38E-02	1.65E-02	3.49E-02
Z-value	8.76E+00	-9.07E+00	-2.55E+00	-7.68E+00	9.50E+00	-1.49	10.12
P	1.97×10^{-18}	1.18×10^{-19}	1.08×10^{-02}	1.65×10^{-14}	2.01×10^{-21}	1.37×10^{-01}	4.50×10^{-24}
N patients in upper of cutoff50	126	389	7	209	200	1	174
N patients in lower of cutoff50	446	186	39	165	374	25	314
N severe patients upper of cutoff50	98	86	3	65	133	0	116
N severe patients lower of cutoff50	110	121	28	112	75	2	69
N other patient in upper of cutoff50	28	303	4	144	67	1	58
N other patient in lower of cutoff50	336	65	11	53	299	23	245
**RR**	**3.15**	**0.34**	**0.60**	**0.46**	**3.32**	**3.40**	**3.03**
95%-CI	[2.6153;3.8025]	[0.2743;0.4210]	[0.2482;1.4359]	[0.3650;0.5752]	[2.6480;4.1529]	[0.2652;43.5901]	[2.4023;3.8314]
%W(common)	3.3	11	0.6	8.4	3.5	0	3.3
test statistic	1.17E+02	9.89E+01		4.86E+01	1.20E+02	1.08E-30	9.31E+01
df	1	1	1	1	1	1	1
p value 1s	1.12×10^{-27}	1.35×10^{-23}	1.43×10^{-01}	1.60×10^{-12}	3.74×10^{-28}	5.00×10^{-01}	2.47×10^{-22}
p value 2s	2.24×10^{-27}	2.69×10^{-23}	2.86×10^{-01}	3.19×10^{-12}	7.49×10^{-28}	$1.00\times10^{+00}$	4.93×10^{-22}
R score with Severity of Covid19	0.42	-0.41	-0.42	-0.43	0.44	-0.47	0.52

P(pearson) with Severity of Covid19	4.51×10^{-308}	4.51×10^{-308}	3.58×10^{-03}	4.51×10^{-308}	4.51×10^{-308}	1.56×10^{-02}	4.51×10^{-308}

The Meta analytical method, such as: the Mantel-Haenszel method, the restricted maximum-likelihood estimator for tauX2, the Q-profile method for confidence interval of tauX2 and tau, the continuity correction of 0.5 in studies with zero cell frequencies, we found in **the Moderate – Severe group for 37 factors (lower part of table)**: RR=1.28 [1.22;1.33] (z=11.23, *P*<.00001), tauX2=0.5394 [0.3239;0.9054], tau=0.7344 [0.5691;0.9515], IX2=98.0% [97.7%;98.3%], H=7.15 [6.65;7.70]. Test of heterogeneity: Q=1842.55, d.f. = 36, *P* <.05. The Outcome + : severe, the Outcome -: moderate
