# Supplementary material for: Multidimensional Machine Learning for Assessing Parameters Associated With COVID-19 in Vietnam: Validation Study
Source: JMIR Form Res. 2023 Feb 16;7:e42895. doi: 10.2196/42895 (PMC9937111; doi:10.2196/42895)
Supplement: Multimedia Appendix 16 [file formative_v7i1e42895_app16.pdf]

Multimedia Appendix 16. Results of clustering imputation involving 66 parameters (linkage model: complete).

ALL GROUPS			MILD			MODERATE			SEVERE		
INDEX	Number clusters	Value Index	INDEX	Number clusters	Value Index	INDEX	Number clusters	Value Index	INDEX	Number clusters	Value Index
kl	3	2.90	*kl*	59	97.69	*kl*	5	5.31	*kl*	2	3.16
ch	41	28.44	*ch*	59	3153.89	*ch*	63	3268.76	*ch*	2	5.80
hartigan	41	Inf	*hartigan*	59	Inf	*hartigan*	63	Inf	*hartigan*	60	Inf
cindex	41	0.00	*cindex*	59	0.00	*cindex*	63	0.00	*cindex*	2	0.45
db	41	0.11	*db*	59	0.01	*db*	63	0.01	*db*	60	0.37
silhouette	41	0.98	*silhouette*	59	1.00	*silhouette*	63	1.00	*silhouette*	60	0.97
duda	5	0.97	*duda*	2	0.92	*duda*	5	0.89	*duda*	2	0.95
pseudot2	5	0.03	*pseudot2*	2	4.70	*pseudot2*	5	2.14	*pseudot2*	2	2.28
ratkowsky	3	0.30	*ratkowsky*	4	0.23	*beale*	18	-1.90	*beale*	6	-2.88
ball	3	29.45	*ball*	3	85.77	*ratkowsky*	4	0.26	*ratkowsky*	13	0.15
ptbiserial	2	0.58	*ptbiserial*	2	0.55	*ball*	3	28.26	*ball*	3	39.26
gap	2	-0.31	*gap*	2	-0.18	*ptbiserial*	5	0.59	*ptbiserial*	11	0.49
mcclain	2	0.03	*mcclain*	2	0.05	*gap*	2	0.14	*gap*	2	0.31
gamma	38	1.00	*gamma*	47	1.00	*mcclain*	2	0.89	*mcclain*	2	0.49
gplus	38	0.00	*gplus*	47	0.00	*gamma*	48	1.00	*gamma*	59	1.00
tau	4	143.44	*tau*	9	248.99	*gplus*	48	0.00	*gplus*	59	0.00
dunn	39	1.43	*dunn*	59	9.89	*tau*	5	279.64	*tau*	4	273.61

sdindex	32	1.86	*sdindex*	44	1.03	*dunn*	63	2.12	*dunn*	60	1.02
sdbw	41	0.00	*sdbw*	59	0.00	*sdindex*	49	2.36	*sdindex*	60	0.88
						sdbw	63	0.00	*sdbw*	60	0.01

Abbreviations of clustering index: *CH* (Calinski and Harabasz 1974), *CCC* (Sarle 1983), Pseudot2 (Duda and Hart 1973), *KL* (Krzanowski and Lai 1988), *Gamma* (Baker and Hubert 1975), *Gap* (Tibshirani et al. 2001), *Silhouette* (Rousseeuw 1987), *Hartigan* (Hartigan 1975), *Cindex* (Hubert and Levin 1976), *DB* (Davies and Bouldin 1979), *Ratkowsky* (Ratkowsky and Lance 1978), *Scott* (Scott and Symons 1971), *Marriot* (Marriot 1971), *Ball* (Ball and Hall 1965), *Trcovw* (Milligan and Cooper 1985), *Tracew* (Milligan and Cooper 1985), *Friedman* (Friedman and Rubin 1967), *Rubin* (Friedman and Rubin 1967), *Dunn* (Dunn 1974).
