# Supplementary material for: Multidimensional Machine Learning for Assessing Parameters Associated With COVID-19 in Vietnam: Validation Study
Source: JMIR Form Res. 2023 Feb 16;7:e42895. doi: 10.2196/42895 (PMC9937111; doi:10.2196/42895)
Supplement: Multimedia Appendix 17 [file formative_v7i1e42895_app17.pdf]

Multimedia Appendix 17. Results of clustering imputation involving 43 parameters for all groups (selected from the second correlation test) and 41 parameters for each group (selected from the relative risk and risk ratio *P* value; linkage model: complete and average).

	**All three group**				**Mild group**				**Moderate group**				**Severe group**			
	**Linkage Model: average**		**Linkage Model: complete**		**Linkage Model: average**		**Linkage Model: complete**		**Linkage Model: average**		**Linkage Model: complete**		**Linkage Model: average**		**Linkage Model: complete**	
	**Number clusters**	**Value Index**	**Number clusters**	**Value Index**	**Number clusters**	**Value Index**	**Number clusters**	**Value Index**	**Number clusters**	**Value Index**	**Number clusters**	**Value Index**	**Number clusters**	**Value Index**	**Number clusters**	**Value Index**
kl	**5**	**9.5283**	9	2.8721	40	69.1061	40	69.1061	40	5.3598	40	5.3598	4	7.6545	40	6.744
ch	43	28.9343	43	28.9343	40	1965.8037	40	1965.8037	40	94.7927	40	94.7927	40	392.9372	40	392.9372
hartigan	43	Inf	43	Inf	40	Inf	40	Inf	40	Inf	40	Inf	40	Inf	40	Inf
cindex	43	0	43	0	40	0	40	0	40	0	40	0	40	0	40	0
db	43	0.1076	43	0.1076	40	0.0112	40	0.0112	40	0.0565	40	0.0565	40	0.0269	40	0.0269
silhouette	43	0.9837	43	0.9837	40	0.9983	40	0.9983	40	0.9902	40	0.9902	40	0.9955	40	0.9955
duda	1	0.9935	1	0.9935	1	0.9971	1	0.9971	1	0.9978	1	0.9978	1	1.0007	3	0.9217
pseudot2	1	0.2758	1	0.2758	1	0.1131	1	0.1131	1	0.0845	1	0.0845	1	-0.0287	3	0.17
ratkowsky	**5**	**0.2786**	**4**	**0.2979**	**6**	**0.2666**	**3**	**0.286**	**5**	0.2949	**4**	**0.3182**	**4**	0.2656	5	0.2534
ball	2	81.3622	2	81.3622	2	107.8555	2	107.8555	2	132.9104	2	132.9104	2	59.1595	2	63.5336
ptbiserial	2	0.5921	2	0.5921	**5**	0.6336	5	0.5641	**4**	0.6998	**2**	0.5814	4	0.5765	6	0.5539
gap	1	0.9345	1	0.9345	1	0.6881	1	0.6881	1	0.5396	1	0.5396	1	0.9505	1	0.9505
mcclain	2	0.0261	2	0.0261	2	0.0324	2	0.0324	2	0.0279	2	0.0279	2	0.0396	2	0.6744
gamma	40	1	40	1	32	1	32	1	37	1	37	1	35	1	35	1
gplus	40	0	40	0	32	0	32	0	37	0	37	0	35	0	35	0
tau	**6**	**162.26**	**4**	**147.3932**	**6**	**147.9378**	**5**	**138.5183**	**5**	146.3598	**5**	138.4915	**4**	141.0598	6	129.0244
dunn	41	1.4578	41	1.4578	40	8.3182	40	8.3182	40	2.3168	40	2.3168	40	2.5988	40	2.5988

sdindex	34	1.8238	33	1.9121	30	1.3027	33	1.378	12	1.3571	33	1.5111	33	1.4268	33	1.4268
sdbw	43	0.0007	43	0.0007	40	0	40	0	40	0.0003	40	0.0003	40	0.0001	40	0.0001

Abbreviations of clustering index: *CH* (Calinski and Harabasz 1974), *CCC* (Sarle 1983), Pseudot2 (Duda and Hart 1973), *KL* (Krzanowski and Lai 1988), *Gamma* (Baker and Hubert 1975), *Gap* (Tibshirani et al. 2001), *Silhouette* (Rousseeuw 1987), *Hartigan* (Hartigan 1975), *Cindex* (Hubert and Levin 1976), *DB* (Davies and Bouldin 1979), *Ratkowsky* (Ratkowsky and Lance 1978), *Scott* (Scott and Symons 1971), *Marriot* (Marriot 1971), *Ball* (Ball and Hall 1965), *Trcovw* (Milligan and Cooper 1985), *Tracew* (Milligan and Cooper 1985), *Friedman* (Friedman and Rubin 1967), *Rubin* (Friedman and Rubin 1967), *Dunn* (Dunn 1974).
